# Disrupting Notch signaling related HES1 in myeloid cells reinvigorates antitumor T cell responses

**DOI:** 10.1186/s40164-024-00588-2

**Published:** 2024-12-19

**Authors:** Myung Sup Kim, Hyeokgu Kang, Jung-Hwan Baek, Moon-Gyu Cho, Eun Joo Chung, Seok-Jun Kim, Joon-Yong Chung, Kyung-Hee Chun

**Affiliations:** 1 Department of Biochemistry & Molecular Biology, Graduate School of Medical Science, Brain Korea 21 Project, Yonsei University College of Medicine, Seodaemun-gu, Seoul, 03722 Republic of Korea; 2 Radiation Oncology Branch, Center for Cancer Research, National Cancer Institute, National Institutes of Health, Bethesda, MD 20892 USA; 3 Department of Biomedical Science, Chosun University, Gwangju, 61452 Republic of Korea; 4 Department of Integrative Biological Sciences & BK21 FOUR Educational Research Group for Age-Associated Disorder Control Technology, Chosun University, Gwangju, 61452 Republic of Korea; 5 Institute of Well-Aging Medicare & Chosun University G-LAMP Project Group, Chosun University, Gwangju, 61452 Republic of Korea; 6 Molecular Imaging Branch, Center for Cancer Research, National Cancer Institute, National Institutes of Health, Bethesda, MD 20892 USA; 7 Institute for Bio-medical Convergence Science and Technology, Yonsei University, Seodaemun-gu, Seoul, 03722 Republic of Korea; 8 Affiliate Faculty, Pohang University of Science and Technology, Pohang, 37673 Republic of Korea

## Abstract

**Background:**

Tumor-associated macrophages (TAMs) are immunosuppressive cells within the tumor microenvironment (TME) that hinder anti-tumor immunity. Notch signaling is a pathway crucial for TAM differentiation and function. Here, we investigate the role of HES1, a downstream target of Notch signaling, in TAM-mediated immunosuppression and explore its potential as a target for cancer immunotherapy.

**Methods:**

In this work, we constructed conditional *Hes1* knockout mice to selectively delete *Hes1* in TAMs. We further analyzed the TME composition, T cell infiltration and activation, and anti-tumor effects in these mice, both alone and in combination with PD-1 checkpoint blockade.

**Results:**

Our study showed that expression levels of Notch target *Hes1* were increase in TAMs and mice with conditional knockout of *Hes1* gene in TAMs exhibited decreased tumor growth, with increased infiltration and activation of cytotoxic T cells in tumors. Expression of tumor promoting factors was critically altered in *Hes1*-conditional KO TAMs, leading to the improved tumor microenvironment. Notably, arginase-1 expression was decreased in *Hes1-*conditional KO mice. Arg1 is known to deplete arginine and deactivate T cells in the TME. Administration of anti-PD-1 monoclonal antibody inhibited tumor growth to a greater extent in *Hes1-*conditional KO mice than in WT mice.

**Conclusions:**

We identified a pivotal role for the Notch signaling pathway in shaping TAM function, suggesting that T-cell dysfunction in the TME is caused when the Notch target, HES1, in TAMs is upregulated by tumor-associated factors (TAFs), which, in turn, increases the expression of arginase-1. Targeting HES1 in TAMs appears to be a promising strategy for cancer immunotherapy.

**Supplementary Information:**

The online version contains supplementary material available at 10.1186/s40164-024-00588-2.

## Background

Although considerable progress has been made in cancer treatment, resistance to therapy remains a major challenge. Both tumor progression and resistance to therapy are promoted by the tumor microenvironment (TME) elements [1]. Mainly, tumor-associated macrophages (TAMs), derived from circulating monocytes, contribute to tumor growth and metastasis [2–4]. Interestingly, TAMs play important roles in shaping the TME by impairing T cell function and metabolism [5–7]. TAMs recruit immunosuppressive cells such as regulatory T cells (Tregs) and myeloid-derived suppressor cells (MDSCs), and inhibit the function of anti-tumor immune cells such as CD8^+^ T cells and natural killer cells [8–10]. Furthermore, TAMs can disrupt the metabolism of cytotoxic T cells, making less effective at eradicating tumor cells by depleting the amino acid arginine from the TME, which is essential for T cell activation and proliferation [11, 12]. TAMs also cause metabolic starvation of T cells through the production of immunosuppressive metabolites by the indoleamine-pyrrole 2,3-dioxygenase 1/2 (IDO1/2) pathway [13–15].

It was previously reported that TAMs differ from tissue macrophages in their origins, characteristics, and function [16–18]. Notch signaling mediates TAM differentiation from monocytes and direct interactions between TAMs and cancer cells [19, 20]. This may present challenges for therapeutic targeting of TAMs [21, 22], as Notch signaling is essential for cellular proliferation and differentiation; moreover, it modulates tumor immune responses, including T-cell differentiation and maturation [23–25]. Among Notch signaling targets, HES1 is one of the most highly upregulated genes in TAMs [19, 26]. It plays a central role in the maintenance of stemness and self-renewal in cancer stem cells, and its upregulation is associated with poor prognosis in several cancer types [27–29].

Driven by theses insights, we aimed to elucidate the molecular mechanism by which Notch signaling in TAMs regulates tumor growth and how HES1 deficiency alters the TME into an immunostimulatory state to assess the potential of HES1 targeting for novel cancer immunotherapy strategies.

## Methods

### Animals

Myeloid-specific *Hes1*-KO C57BL/6 J mice were generated by mating *Hes1*^*fl/fl*^ mice (kindly provided by Young-Yun Kong, Seoul National University, Seoul, Korea) with LysM-Cre mice (kindly provided by Heung Kyu Lee, Korea Advanced Institute of Science & Technology, Daejeon, Korea). MMTV-PyMT mice (kindly provided by Han-Woong Lee, Yonsei University, Seoul, Korea) were crossed with LysM-*Hes1*^*fl/fl*^. Age- and sex-matched cohorts of female (8–12-week-old) and male (6–12-week-old) littermates were used in subcutaneous tumor graft experiments. Appropriate littermate controls were used in all experiments. All animal experiments were approved by the Institutional Review Board of the Yonsei University College of Medicine. The mice were housed in pathogen-free facilities, in a 12-h light/dark cycle in ventilated cages, with ad libitum access to chow and water in accordance with the guidelines for the Care and Use of Laboratory Animals (2018–0289).

### Cell culture, transfection, and reagents

The TC-1 lung cancer cell line (from C57BL/6 mice) was kindly provided by Sang-Jun Ha (Yonsei University). The EO771 breast cancer cell line (from female C57BL/6 mice) was provided by Kyu Lim (Chungnam National University, Daejeon, Korea). The MC-38 adenocarcinoma cell line (from female C57BL/6 mice), B16-F10 melanoma cell line (from male C57BL/6 mice), 4T-1 mammary carcinoma cell line (from BALB/c mice), CT-26 colon carcinoma cell line (from BALB/c mice), L-929 fibroblast line (from C3H/An mice), HEK-293, and RAW264.7 cell line (from male BALB/C mice) were purchased from the American Type Culture Collection (Manassas, VA, USA). Cells were maintained in RPMI 1640 or DMEM (Welgene, Kyungsan, Korea) supplemented with 10% FBS and 1% penicillin–streptomycin (Invitrogen, Waltham, MA, USA). All cell lines used in the tumor studies were confirmed to be mycoplasma-negative using a MycoAlert Mycoplasma Detection kit (Lonza, Basel, Switzerland). Cells were cultivated at 37 ℃ under 5% CO_2_. Transfection with the HES1 expression vector as well as with siRNAs against *Stat6*, *Rbpj*, and *Hes1* was performed using Lipofectamine 2000 and Lipofectamine RNAiMax (Invitrogen) according to the manufacturer’s instructions. Two siRNAs were used to confirm the results. *Stat6* siRNA #1 (5′-GUGAAAGCCUGGUGGAAAU-3′), *Stat6* siRNA #2 (5′-GAAACAGGCCAGAGAACUA-3′), *Rbpj* siRNA #1 (5′-GCACAGAAGUCUUACGGAA-3′), *Rbpj* siRNA #2 (5′-GCAUGUAGAAGGAGGGAAU-3′), *Hes1* siRNA #1 (5′-UGAAAGUCUAAGCCAACUGAATT-3′) and *Hes1* siRNA #2 (5′-CCAGCCAGUGUCAACACGACATT-3′) were purchased from GenePharma (Shanghai, China). *Mus musculus Hes1* ORF sequences were amplified using high-fidelity PCR from full-length cDNA using primers mHes1-Fw (*Sal*I) 5′-AAATTGTCGACGCCGCCACCATGGATTACAAGGATGACGACGATAAGAGCGC-3′ and mHes1-Rev (*Not*I) 5′-AATTTGCGGCCGCTCAGTTCCGCCACGGTCTCCAC-3′ and ligated into the pCMV-Sport6 vector (Addgene). DH5α (NEB) cells were used to expand the plasmid, and colonies were selected using 100 µg/mL ampicillin. Plasmid DNA was purified and sequenced to verify the construct. Recombinant murine IL-4 (#214-14), IL-10 (#210-10), IL-13 (#210-13), IFN-γ (#315-05), and macrophage colony-stimulating factor (M-CSF) (#315-02) were purchased from Peprotech (Rocky Hill, NJ, USA). LPS (L3024) was obtained from Sigma-Aldrich (St. Louis, MO, USA). Transwell plates (#3422) were obtained from Corning (Corning, NY, USA).

### Total RNA isolation, reverse transcription, and qRT-PCR

Total RNA was extracted using TRIzol Reagent (Invitrogen) following the manufacturer’s instructions. cDNA was synthesized using RT-PCR Master Mix (TOYOBO, Osaka, Japan) from 3–5 μg RNA. qRT-PCR was performed using SYBR premix Ex Taq (Takara, Kusatsu, Shiga, Japan) on an QuantStudio 3 (Applied Biosystems, Waltham, MA, USA). *β-actin* was used for normalization. RNA quality assessment prior to sequencing was performed using a 2100 Bioanalyzer (Agilent Technologies, Santa Clara, CA, USA).

### Western blotting

Cells were lysed using 20 mM Tris HCl, 150 mM NaCl, 1% Triton X-100, 1.5% MgCl_2_, 1 mM EDTA, 1 mM Na_2_VO_4_, 1 mM phenylmethylsulfonyl fluoride and protease inhibitor cocktail (Xpert Protease Inhibitor Cocktail Solution, GenDEPOT, Katy, TX, USA), pH 7.5. Lysates were briefly vortexed and cleared by centrifugation at 13,200 rpm for 20 min at 4 ℃. Supernatants were transferred to fresh microcentrifuge tubes and protein concentrations were measured using Bradford assay (Thermo Fisher Scientific, Waltham, MA, USA). Proteins (20–50 μg) were separated using SDS-PAGE, electroblotted onto nitrocellulose membranes (Amersham Biosciences, Woburn, MA, USA), and then blocked in 5% skim milk powder and 0.05% Tween-20 in PBS (PBST). Primary antibodies were incubated with blots at dilutions of 1 in 1000–3000 in minimal volumes of 5% BSA in PBST for 1 h at room temperature or overnight at 4 °C. Membranes were incubated with horseradish peroxidase–conjugated anti-mouse, anti-rabbit, or anti-goat secondary antibodies (Bethyl Laboratories, Montgomery, TX, USA) at 1:10,000 dilution in 5% skim milk powder in PBST for 1 h at room temperature. Anti-HES1, anti-HEY1, anti-PPARG, anti-STAT3, anti-p-STAT3 (Y705), anti-CD206, anti-GAPDH, anti-p65, anti-p-p65 (S536), anti-CD80, anti-CD86, anti-RBPJ, anti-β-actin, anti-C/EBPA, anti-C/EBPB, anti-AKT, anti-p-AKT (Th308), anti-p-AKT (S473), anti-IKBA, anti-p-IKBA (S32), anti-JNK, anti-p-JNK (Thr183/Tyr185), anti-p38, and anti-p-p38 (Thr180/Tyr182) antibodies were purchased from Santa Cruz Biotechnology (Dallas, TX, USA). Anti-p70S6K, anti-p-S6K (Th389), anti-p-S6K (Th421), anti-STAT1, anti-p-STAT1 (Y701), anti-IRF4, anti-IRF9, anti-SHP2, anti-HIF1A, anti-STAT6, anti-p-STAT6 (Y641), anti-iNOS, anti-ERK1/2, and anti-p-ERK1/2 (Thr202/Tyr204) antibodies were purchased from Cell Signaling Technology (Danvers, MA, USA). Anti-NICD, anti-PDL1, and anti-ARG1 antibodies were obtained from Abcam (Cambridge, UK). The membranes were washed 3 times for 10 min each. A FUSION SOLO imager (Vilber, Marne-la-Vallée, France) was used for image detection according to the manufacturer’s instructions. β-Actin or GAPDH were used as loading controls.

### Syngeneic tumor models

TC-1 cells (1.0 × 10^5^), B16F10 cells (2.5 × 10^5^), EO771 cells (5.0 × 10^5^), MC-38 cells (5.0 × 10^5^), 4T-1 cells (1.0 × 10^6^), or CT-26 cells (1.0 × 10^6^) were injected subcutaneously into the middle lower back of mice. In accordance with the ethics committee’s guidelines, two subcutaneous injections were performed, and the tumor was kept from growing so that the sum of the major axes did not exceed 20 mm. Experimenters were blinded to the genotype. Growth was monitored by measuring tumor size using a digital caliper. Volume was estimated using the formula: length × width^2^ × 0.5. Tumor-infiltrating immune cells were isolated by harvesting tumors 14–18 days after implantation following euthanasia.

### Myeloid cell depletion

To achieve in vivo depletion of CSF1R^+^ cells, cohorts of mice were administered purified anti-CSF1R rat mAb (clone AFS98; BioXCell, Lebanon, NH, USA) or control isotype immunoglobulin (IgG2a) via intraperitoneal injection (400 μg per mouse). The treatment regimen consisted of an initial dose 1 week prior to tumor cell injection, followed by treatments at intervals of 6 d. To specifically deplete Ly-6G^+^ cells in vivo, purified anti-Ly-6G rat mAb (clone 1A8, BioXCell) or control isotype (clone 2A3, BioXCell) was administered to separate cohorts of mice. Antibodies were administered via intraperitoneal injection (300 μg per mouse) 5 d prior to tumor cell injection and at 48-h intervals thereafter.

### T-cell depletion

Mice were treated with 200 μg rat-anti-CD4 (clone GK1.5, BioXCell), rat-anti-CD8 (clone 2.43, BioXCell), or rat IgG2b anti-KLH isotype control (clone LTF2, BioXCell) antibodies diluted in *InVivo* Pure pH 7.0 Dilution Buffer (IP0070, BioXCell) via intraperitoneal injection, with a treatment schedule of every three days commencing one day before the tumor cell injection.

### T cell proliferation assay

CD4^+^ or CD8^+^ T cells were isolated from the spleens of wildtype C57BL/6 mice the using CD4^+^ (Miltenyi Biotec, 130-104-454) or CD8^+^ (Miltenyi Biotec, 130-104-075) T cell isolation kit, respectively. The isolated T cells were labeled with 5 μM CellTracer™ Violet (CTV) cell proliferation kit (ThermoFisher, C34557). Following isolation, the T cells were either left unstimulated or were activated using plate-bound anti-CD3 (2 μg/mL) and anti-CD28 (1 μg/mL) antibodies. Cocultures were established with IL-4 (20 ng/ml) or EO771 CM treated BMDMs from wildtype or *Hes1* cKO mice at a cell ratio of 1:3 (0.3 × 10^4^ BMDM: 1 × 10^5^ T cells). After three days of culture, cells were harvested, stained with CD4 or CD8 antibodies, and analyzed by flow cytometry.

### Tumor digestion

Tumors were harvested, minced, and further dissociated using a gentleMACS Dissociator (#130-093-235, Miltenyi Biotec, Bergisch Gladbach, Germany), then digested with a MACS Miltenyi Tumor Dissociation Kit for mice (#130-096-730, Miltenyi Biotec) according to the manufacturer’s instructions. The resulting tumor cells were washed with DMEM and dissociated by gently pushing and pulling a syringe plunger and pipetting. The cell suspension was passed through a 70-μm cell strainer (SPL Life Science, Pocheon-si, Korea) and red blood cells were lysed using RBC Lysis Solution (Biosesang, Seong-nam, Korea). The tumor cells were washed again and collected from the 40−80% interface of a Percoll gradient.

### Flow cytometry

Single-cell suspensions were prepared from spleen, bone marrow, and tumor tissues of normal and tumor-bearing mice. Spleens and bone marrow cells were labeled using fluorochrome-conjugated antibodies against CD3 (clone 17A2), CD4 (clone GK1.5), CD8 (clone 53-6.7), CD45R/B220 (clone RA3-6B2), Ly-6G/Ly-6C (Gr1) (clone RB6-8C5), Ly-6A/E (Sca-1) (clone D7), and CD117 (c-kit) (clone 2B8), as well as isotype-matched IgG controls (BioLegend, San Diego, CA, USA). Tumor cells were prepared as described above before being resuspended in PBS containing 2% FBS and 2 mM EDTA for flow cytometry. A LIVE/DEAD Fixable Near-IR Dead Cell Stain Kit (L-10119, Invitrogen) was used with cells in combination with anti-mouse CD16/CD32 Fc blocker antibody (#14-0161-81, Invitrogen) for 15 min on ice in the dark. Cells were washed and incubated with the fluorochrome-conjugated antibodies (BioLegend) against CD45 (clone 30-F11), CD4 (clone GK1.5), CD8 (clone 53-6.7), CD45R/B220 (clone RA3-6B2), NK-1.1 (clone PK136), F4/80 (clone BM8), CD11b (clone M1/70), Ly-6C (clone HK1.4), Ly-6G (clone 1A8), CD11c (clone N418), I-A/I-E (clone M5/114.15.2), CD86 (clone GL-1), CD80 (clone 16-10A1), and CD206 (clone C068C2) at the manufacturer’s recommended dilution for 30 min on ice in the dark. For samples requiring intracellular staining, cells were fixed with Fixation/Permeabilization Diluent (#00-5223-56, Invitrogen) for 30 min at RT, washed twice with Permeabilization Buffer (#00-8333-56, Invitrogen), and incubated with Alexa Fluor-488-conjugated antibodies against HES1 (clone EPR4226), FOXP3 (clone FJK-16 s), and ARG1 (clone A1exF5) in permeabilization buffer for 30 min at room temperature. For assessment of cytokine production by tumor-infiltrating lymphocytes, antibodies against IFN-γ (clone XMG1.2), granzyme B (clone GB11), IL-12/p40 (clone C15.6), and TNF-α (clone MP6-XT22) and purified cells were incubated with cell activation cocktail containing brefeldin A (#423303, Biolegend, San Diego, CA, USA) for 4 h at 37 ℃ under 5% CO_2_. Surface staining was carried out as described above; cells were fixed and permeabilized using Cyto-Fast Fix/Perm Buffer set (#426803, Biolegend) and intracellular staining was carried out according to the manufacturer’s instructions. Following staining, cells were washed again with permeabilization buffer, then with PBS, and resuspended in FACS Buffer for flow cytometric analysis on the BD LSRFortessa cell Analyzer (BD Biosciences, San Jose, CA, USA) at the Flow Cytometry Core of the Avison Biomedical Research Center in Yonsei College of Medicine. A total of 10,000–1,000,000 cells were analyzed per sample per mouse using BD FACS Diva Software. Data were analyzed using the FlowJo software. For TAM sorting, single-cell suspensions of tumor tissues were stained with fluorescently-labeled antibodies and sorted using a FACSAria flow cytometer (BD Biosciences). Live/Dead^−^CD45^+^CD11b^+^F4/80^+^ cells were collected for gene expression analysis.

### Preparation of BMDMs

Cohorts of male mice aged 6–12 weeks were euthanized and both femur and tibia were dissected free of adherent tissue. Bone marrow cells from the femur and tibia were subjected to red blood cell lysis using red blood cell lysis buffer, and surviving cells were cultured for 6 d in differentiation medium comprising Dulbecco’s minimum essential (DME) medium supplemented with 30% L929 cell culture supernatant, 20% heat-inactivated FBS, and 1% penicillin/streptomycin [30]. On day 3, 5 mL medium was added. Cells were harvested using cold PBS, washed, resuspended in DMEM supplemented with 10% FBS and 1% penicillin/streptomycin, and used at a density of 2−10 × 10^5^/mL in subsequent experiments. To confirm the development of BMDMs, antibodies (BioLegend) against CD11b (clone M1/70), F4/80 (clone BM8), and MHC class II (I-A/I-E; clone M5/114.115.2) were used. Cells were analyzed using a BD LSRFortessa Cell Analyzer or GUAVA easyCyte 5HT (Cytek Biosciences, Fremont, CA, USA) flow cytometer. Appropriate gates were set for forward and side scatter in analyzing each population. To polarize the BMDMs, classical or alternative activation was induced by culturing the cells for 1−2 d. Classical activation was induced with 20 ng/mL of recombinant mouse IFN-γ and 100 ng/ml LPS, while alternative activation was induced with 20 ng/mL recombinant mouse IL-4. To rule out unintended effects of L929 cell culture supernatant on BMDMs, BMDMs were differentiated using recombinant M-CSF for comparison.

### L929- and tumor-CM

L929 cells were maintained in DMEM supplemented with 10% FBS as the biological source of CSF-1 for BMDM culture. L929 cells were plated at a density of 4.7 × 10^5^ cells/well in a 75-cm^2^ flask containing 55 mL 10% FBS/DMEM. Cells were placed in a humidified incubator with 5% CO_2_ at 37 ℃ for 7 d. The supernatant was collected and filtered (0.45 μm). Supernatants were prepared using B16-F10, EO771, MC-38, and TC-1 cells. Each cell line was grown in DMEM-complete medium or RPMI complete medium in a 75-cm^2^ flask. Supernatants were collected on days 1, 2, 3, and 4 and filtered using a sterile 33-mm filter (#PR03691, Millex-GV, Merck, Darmstadt, Germany) using a 10-mL syringe. Supernatants were stored at − 20 ℃ until use.

### Transwell coculture

Bone marrow cells were collected from the femur and tibia of male C57BL/6 mice and differentiated into BMDMs using a previously described protocol [30]. On day 7, cells were harvested from a 90 × 15 mm Petri dishes (#10090, SPL) in cold PBS, washed, and resuspended in medium (DMEM with 10% heat-inactivated FBS, 1% penicillin/streptomycin, 1% HEPES, and 1% sodium pyruvate). Cells were centrifuged at 1500 rpm for 5 min and the pellet was resuspended in DMEM. BMDMs (1.5 × 10^5^ per well) were plated in the lower compartment of 6.5-mm polycarbonate transwell inserts with a pore size of 0.4 μm (#3413, Corning) in maintenance medium one day prior to seeding of other cells. The next day, the medium was replaced with fresh medium 1 h before adding B16F10, EO771, MC-38, TC-1 or HEK-293 cells (1.5 × 10^5^ per well), into the transwell inserts. Cells were co-cultured for 1−3 d in a humidified chamber at 37 °C. Control wells contained only BMDMs in the lower compartment with medium.

### ChIP

ChIP was performed on 5 × 10^6^ cells using a Pierce Agarose ChIP kit (#26156, Thermo Fisher Scientific) according to the manufacturer’s instructions. Nuclear lysates were precipitated with anti-Hes1 (NBP1-47791, Novus Biologicals, Littleton, CO, USA), anti-STAT6 (#9362, Cell Signaling Technology), or normal rabbit IgG (#sc-2027, Santa Cruz Biotechnology) antibodies overnight at 4 °C. The recovered DNA was amplified using qRT-PCR. HES1 binding sites were predicted by the JASPAR website (https://jaspar.genereg.net/).

### Luciferase assay

The murine *Arg1* promoter sequence was cloned into the pGL3-Basic reporter plasmid (pGL3-Basic-Arg-1) spanning from position − 3200 to + 100 bp and validated by sequencing. Using Lipofectamine 2000, cells were co-transfected with the *Arg1* reporter plasmid and either *Hes1* expression plasmid (100 ng) or *Hes1* siRNA together with pCMV-β-Gal plasmid. After 18 h, cells were stimulated with IL-4 (20 ng/mL) or EO771 CM for the times indicated. The murine *Hes1* reporter plasmids pHes1 (467)-luc (Addgene plasmid #41723; http://n2t.net/addgene:41723; RRID: Addgene_41723) and pHes1 (467 RBPj (-))-luc (Addgene plasmid #43805; http://n2t.net/addgene:43805; RRID: Addgene_43805) were gifts from Ryoichiro Kageyama (RIKEN Center for Brain Science, Kyoto University). pGL2 basic, pHes1 (467)-luc, or pHes1 (467 RBPj (-))-luc, together with pCMV-β-Gal, were co-transfected into BMDMs using Lipofectamine 2000. At 18 h after transfection, cells were stimulated with IL-4 or tumor supernatants for 6 h. Cells were washed with PBS and lysed with Cell Culture Lysis Reagent (Promega, Madison, WI, USA), and luciferase activity was normalized to β-galactosidase activity in the cell lysate.

### PD-1 checkpoint blockade

Once tumors became palpable, mice were randomly assigned to treatment groups and administered with 200 μg antibodies. The treatment, either an isotype control (clone 2A3, cat. #BE0089, BioXCell) or anti-PD-1 antibody (clone RMP1-14, cat. #BE0146, BioXCell), was administered intraperitoneally every three days, starting as soon as the tumors began to form.

### QuantSeq 3′ mRNA sequencing

Reads were aligned to the genome assembly sequence or the representative transcript sequences using Bowtie2 (version 2.3.4.3). The resulting alignment file was used for transcript assembly, estimation of abundance, and differential gene expression analyses. Differentially expressed genes were determined based on counts from unique and multiple alignments using coverage in Bedtools (version 2.26). The read count data were normalized using the quantile normalization method with EdgeR within R version 4.0.3 (R development Core Team, 2016) using Bioconductor. Gene sets were obtained from the Molecular Signature Database (version 7.0). Gene classification was performed by DAVID (http://david.abcc.ncifcrg.gov/).

### Immunohistochemistry

Staining was performed on 5-μm-thick formalin-fixed paraffin-embedded tissue sections. Sections were deparaffinized using xylene and dehydrated using a graded ethanol series. Endogenous peroxidase activity was quenched using 3% H_2_O_2_ for 20 min at room temperature. The sections were subjected to antigen retrieval and incubated with antibodies against CD45, CD11b, CD3e, F4/80, ARG1, CD163, and HES1. Antibody-labeled sites were visualized using 3,3′-diaminobenzidine, lightly counterstained with hematoxylin, dehydrated in ethanol, and cleared in xylene. Negative controls (rabbit IgG and omission of primary antibody) were prepared concurrently. Slides were scanned using an Aperio AT2 digital scanner with a 40 × objective (Leica Biosystems, Wetzlar, Germany). Twenty representative fields were captured from virtual microscopic images, and numbers of positive cells were counted using ImageJ software (National Institutes of Health, Bethesda, MD, USA). Mean numbers of positive cells per unit area (0.71 mm^2^) were calculated from 20 areas for each case.

### Statistical analysis

Data were analyzed using Prism 7.0 (GraphPad Software Inc., San Diego, CA, USA) and are presented as means ± SD or SEM as indicated. Statistical comparisons among multiple treatment groups were conducted using a one-way ANOVA for comparison of the means among groups. Two-tailed unpaired *t*-tests were used for comparisons between two groups. Statistical significance was set at p < 0.05.

## Results

### TAMs exhibit high Hes1 expression

**Fig. 1:**
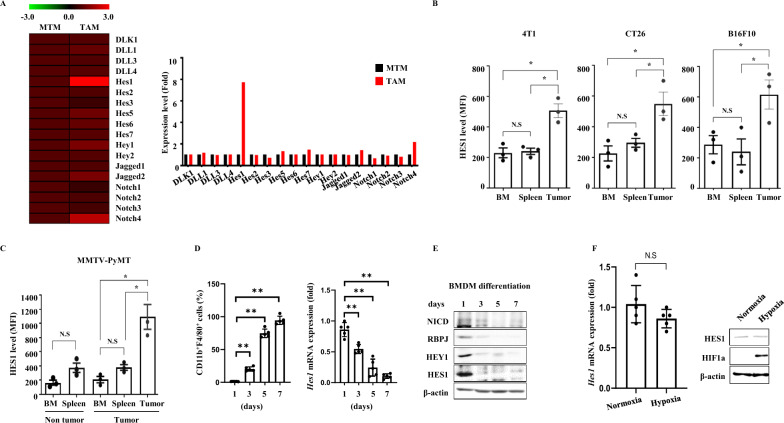
Elevated expression of HES1 in tumor associated macrophages. **A** Gene expression analysis was performed on TAMs and mammary tissue macrophages (MTMs) obtained from 16-week-old female MMTV-PyMT mice (GES56755). Canonical Notch signaling related genes with a p-value threshold lower than 0.05 were considered to be differentially expressed. The fold change was represented using a log scale. **B** The mean fluorescence intensity (MFI) of intracellular HES1 staining was measured in macrophages (CD11b^+^F4/80^+^ double positive) isolated from bone marrow, spleen, and tumors. Mice were subcutaneously injected with 4T1, CT26 or B16F10 cell lines to generate tumor tissues. **C** The intracellular HES1 staining from MMTV-PyMT mice. Macrophages from bone marrow, spleen, and tumors were analyzed by flow cytometry. **D** Expression of HES1 in differentiating macrophages obtained from bone marrows of normal mice. The population of differentiated macrophages were analyzed by flow cytometry based on CD11b^+^F4/80^+^ cells (also see Supplemental Fig. 3). Additionally, *Hes1* mRNA levels during the differentiation of BMDMs were analyzed using qRT-PCR. Relative expression was normalized to the expression level at the beginning of the experiment, designated as ‘day1’. **E** Protein expression levels of HES1 during BMDM differentiation were analyzed by Western blotting. The expression levels of Notch signaling-related factors, including NICD, RBPJ, and HEY1 were also measured over the course of BMDM differentiation. **F** BMDMs were subjected to normoxic (20.9% O_2_) or hypoxic (1% O_2_) conditions for 24 h. The expression of HES1 was measured using both qRT-PCR and Western blotting. β-actin expression was used as the normalization control for both methods. The presented data are expressed as mean ± SEM, with asterisks denoting significant differences (* p < 0.05) determined by the student’s t-test

We investigated changes in gene expression in the Notch signaling pathway during the differentiation of macrophages to TAMs. Publicly available data (GSE56755) showed that *Hes1* was significantly upregulated in TAMs, but not in mammary tissue macrophages (MTMs) from mammary-specific polyomavirus middle T antigen overexpression (MMTV-PyMT) mice (Fig. 1A). To investigate gene expression in the context of tumor development, bone marrow, spleen, and tumor macrophages were sorted to yield CD45^+^CD11b^+^F4/80^+^ cells (Figure S1A-B). When compared with the results seen in bone marrow or splenic macrophages, TAMs from MMTV-PyMT mice exhibited more substantial changes in *Hes1* expression among genes related to Notch signaling (Figure S2). We next measured intracellular HES1 protein levels using flow cytometry. TAMs were collected from subcutaneous tumor-bearing mice and compared with bone marrow and splenic macrophages collected from the same mice. We found that HES1 was highly expressed in TAMs, but not in bone marrow or splenic macrophages in these mice (Fig. 1B). We also confirmed that the expression of HES1 increases concurrently with tumor development using MMTV-PyMT mice (Fig. 1C). By contrast, in the bone marrow of normal mice, HES1 protein levels gradually declined in parallel with *Hes1* mRNA levels toward the end of macrophage differentiation with no apparent defects in bone marrow derived macrophages (BMDMs) differentiation (Fig. 1D, E and Figure S3). HES1 expression can be induced by the transcription factor HIF-1α in a Notch-independent manner during hypoxia [31, 32]. Notably, hypoxia, a common characteristic of the TME, did not induce HES1 expression (Fig. 1F). These data indicate that HES1 is significantly upregulated in TAMs and that its expression increases at tumor onset.

### Tumor cell-secreted soluble factors increase HES1 expression

**Fig. 2:**
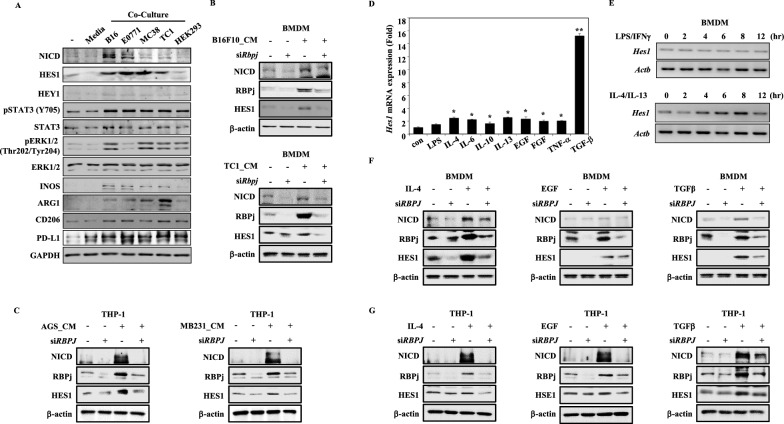
Tumor promoting factors lead to HES1 expression in a Notch signaling pathway-dependent manner. **A** BMDMs were cultured alone or co-cultured with tumor cell lines (B16F10, E0771, MC38, or TC-1) or human embryonic kidney cell line (HEK293). Protein levels of HES1, as well as markers of pro-tumoral macrophage and the Notch signaling pathway were detected by Western blotting. Phosphorylation status of STAT3 was analyzed to confirm the efficiency of co-culture of tumor cells and BMDMs. GAPDH was used as a loading control. **B** BMDMs were transiently transfected with siRNA against *Rbpj* for at least 24 h and treated with CM obtained from B16F10 (skin) or TC-1 (lung) for 8 h. β-actin expression was used as the normalization control. **C** *RBPJ* in THP-1 was knock-down by siRNA for at least 24 h and treated with CMs from AGS (stomach) or MB231 (breast) cell lines for 8 h. β-actin was used as a loading control. **D** BMDMs were exposed to purified cytokines and growth factors known to promote tumor growth, and *Hes1* expression was measured using qRT-PCR. **E** BMDMs were treated to either an inflammatory stimulus (100 ng/ml LPS with 20 ng/ml IFN-γ) or an anti-inflammatory stimulus (20 ng/ml IL-4 with 20 ng/ml IL-13) for the indicated periods. The expression levels of *Hes1* were measured by qRT-PCR. **F** *Rbpj*-knocked down (at least 24 h) BMDMs were treated with 20 ng/ml IL4, 20 ng/ml EGF, or 20 ng/ml TGFβ for 8 h. β-actin was used as a loading control. **G** *RBPJ* was transiently knocked down in THP-1 cells at least for 24 h and treated with 20 ng/ml IL4, 20 ng/ml EGF, or 20 ng/ml TGFβ for 8 h. β-actin was used as a loading control

To assess the effect of tumor-derived soluble factors on macrophage activation and induction of HES1 expression, we used transwell co-culture experiments. Multiple mouse tumor cell lines were co-cultured with BMDMs to identify changes in phenotype. We observed a significant increase in HES1 expression, in parallel with that of pro-tumor macrophage markers, including ARG1, CD206, PD-L1, and Notch signaling-related proteins (Fig. 2A). However, no comparable changes were seen when BMDMs were co-cultured with non-tumor cell lines, including human embryonic kidney (HEK)-293 cells. To understand how HES1 expression in macrophages is activated in response to tumor-promoting conditions, we knocked down *Rbpj* using siRNA, followed by treatment with mouse tumor cell conditioned medium (CM). RBPJ mediates the transcription of genes in the Notch signaling pathway, including the *Hes* and *Hey* gene families. To first determine whether RBPJ regulates *Hes1* expression in macrophages, a luciferase assay was used (Figure S4). On treatment with tumor-CM, enhanced luciferase activity was seen for the promoter sequence of *Hes1* with the RBPJ consensus binding motif. Furthermore, mutations in the RBPJ-binding motif decreased luciferase activity. We also confirmed that HES1 levels were regulated in an RBPJ-dependent manner on treatment with tumor conditioned media from B16F10 or TC-1 (Fig. 2B). Regulation of HES1 expression by RBPJ was also observed in a human macrophage cell line, THP-1 cells, on treatment with tumor conditioned media from AGS or MB231. (Fig. 2C). As tumor conditioned media contain a number of tumor-promoting cytokines and growth factors that suppress immune responses, we investigated *Hes1* expression in response to these stimuli. We found that various tumor-promoting cytokines increased *Hes1* expression (Fig. 2D, E and Figure S5A-B). Consistent with this finding, publicly available RNA-sequencing data (GSE99296) confirmed reduced expression of *Hes1* on treatment of macrophages with LPS and enhanced expression of *Hes1* on treatment with IL-4 (Figure S5C). Tumor-promoting factors, including IL-4, EGF, and TGF-β, induced HES1 expression, but *Rbpj* knockdown attenuated these responses in both murine and human macrophage cell lines (Fig. 2F, G). Thus, HES1 expression is upregulated in response to tumor-derived soluble factors or anti-inflammatory stimuli and this phenotype is regulated by the Notch signaling pathway.

### Myeloid-specific Hes1-KO mice display normal hematopoietic stem cell development

**Fig. 3:**
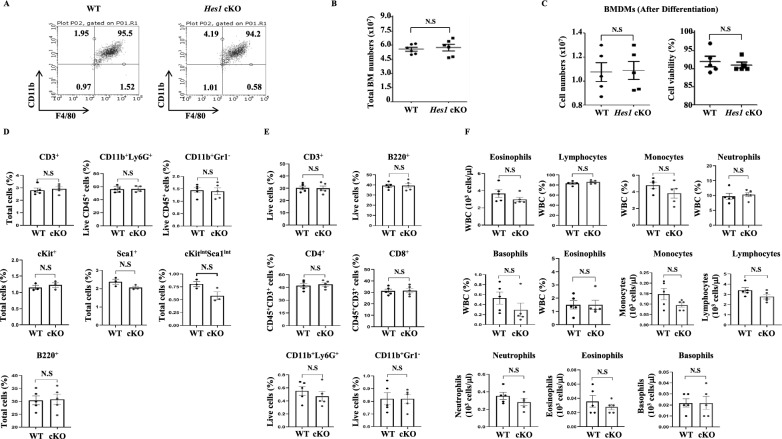
The development of hematopoietic stem cells is normal in mice with myeloid specific *Hes1* knock out. **A** Bone marrow cells from wild-type (WT) or *Hes1* conditional knockout (cKO) mice were cultured and differentiated into BMDMs for 7 days in vitro. On day 7, samples were collected, and macrophages were identified by flow cytometry (CD11b^+^F4/80^+^). Representative flow cytometry plots of fully differentiated BMDMs from WT and *Hes1*-cKO mice are shown. **B** Total number of bone marrow cells from WT and *Hes1* cKO littermates before differentiation. **C** The number of differentiated BMDMs and cell viability after differentiation. **D** Flow cytometry analysis of bone marrow cells for stem cells (cKit^+^, Sca1^+^, or ckit^int^Sca1^int^), lymphocytes (T cells: CD3^+^; B cells: B220^+^) and myeloid cells (neutrophils: CD11b^+^Ly-6G^+^; monocytes/macrophages: CD11b^+^Gr1^−^) in the bone marrow. Six to eight-week-old mice were used. **E** Flow cytometry for lymphocytes (T cells: CD3^+^; B cells: B220^+^), myeloid cells (neutrophils: CD11b^+^Ly-6G^+^; monocytes/macrophages: CD11b^+^Gr1^−^) and T cell subpopulation (CD3^+^CD4^+^ T cells; CD3^+^CD8^+^ T cells) in the spleen. Six to eight-week-old mice were used. **F** The CBC in peripheral blood of six to eight-week-old mice to analyze overall health and conditions depending on *Hes1* deficiency

HES1 is highly expressed in the early stages of T-cell development and is thought to be a potent factor promoting a proliferative state [33, 34]. HES1 is also involved in hematopoietic stem cell differentiation in vitro [20, 26, 35]. To rule out possible adverse effects of *Hes1* deletion in the myeloid lineage (*Hes1*-cKO) on hematopoiesis and T-cell development, we confirmed that there were only negligible differences in the cellularity of differentiated macrophages from bone marrow in *Hes1*-cKO mice compared to that in WT littermates (Fig. 3A–C). We observed no differences in BMDM differentiation ability, cell count before and after differentiation, or viability. We examined whether *Hes1* deletion in the myeloid lineage affects steady-state hematopoiesis and found comparable populations of lymphocytes (B220^+^ B cells and CD3^+^ T cells) and myeloid cells (CD11b^+^Ly6-G^+^ neutrophils and CD11b^+^Gr1^−^ monocytes/macrophages) independent of *Hes1* genotype. Further analysis revealed that *Hes1-*cKO mice showed only marginal differences in their relative frequencies of hematopoietic progenitor cells (cKit^+^, Sca1^+^, and cKit^int^Sca1^int^) (Fig. 3D). HES1 is also expressed in the spleen and plays critical roles in hematopoiesis and immune function [36]. We assessed immune cell populations in the spleen following *Hes1* conditional knockout but found no significant differences (Fig. 3E). Furthermore, we analyzed the complete blood count (CBC) in the peripheral blood of WT or *Hes1-*cKO mice and found no significant differences in any differentiated lineage cells (Fig. 3F). These data indicate that the effect of conditional myeloid KO of *Hes1* on differentiation and normal hematopoiesis is negligible.

### Myeloid-specific *Hes1* KO mice demonstrate suppressed subcutaneous tumor growth

**Fig. 4:**
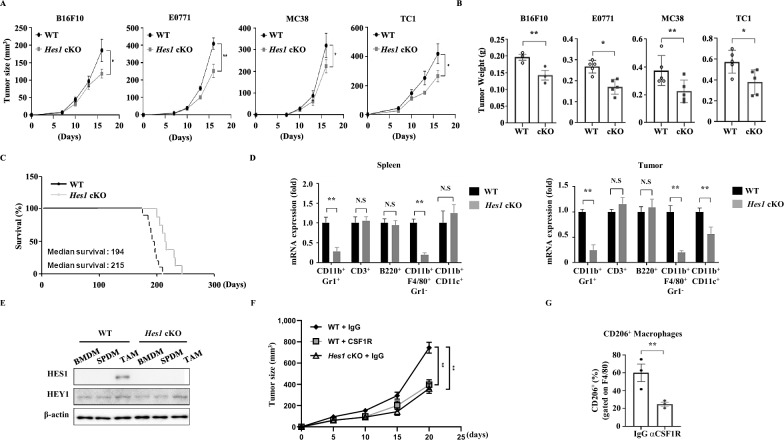
*Hes1*-cKO leads to reduced tumor growth. **A** Six to eight-week-old mice were utilized in the study to evaluate tumor growth in both WT and *Hes1* deficient mice after subcutaneous injection of B16F10, E0771, MC-38 and TC-1 cells. Each group comprised of five WT/*Hes1*-cKO mice. (**B**) Weight of tumors generated in (**A**). **C** Survival of mice bearing tumors in *LysM-Hes1*^+*/*+^ PyMT (n = 10) and *LysM-Hes1*^*fl/fl*^ PyMT mice (n = 14). **D** Different immune cell populations from the spleen and tumor tissue of TC-1 tumor-bearing mice. Immune cells were sorted and followed by qRT-PCR to measure the expression level of *Hes1* in each sorted population. **E** Cells sorted from TC-1 tumor-bearing mice and protein lysates from each sorted population were analyzed by Western blotting. β-actin was used as a loading control. **F** Tumor growth was measured in mice treated with anti-CSF-1R (CSF1R antibody) or an isotype control antibody, as shown in Supplemental Fig. 6B. Each group included two to three tumors per mouse. Six to eight-week-old mice (n = 3) were used for the study. **G** The numbers of CD45^+^Gr1^−^CD11b^+^F4/80^+^CD206^+^ macrophages were quantified in WT mice treated with α-CSF1. The graphs depict the mean ± SEM of data from more than 2 independent experiments

We next examined the impact of myeloid-specific *Hes1* KO on tumor growth by subcutaneous injection of murine melanoma cells (B16F10) into WT and *Hes1*-cKO mice. *Hes1* deletion resulted in substantially reduced tumor growth compared with that in the WT mice. We also injected mice with murine breast (EO771), colon (MC-38), or lung (TC-1) tumor cells and found that *Hes1* deletion resulted in reduced tumor growth in all three models (Fig. 4A). *Hes1* deletion also resulted in a significant reduction in tumor weight (Fig. 4B). We next generated MMTV-PyMT *LysM-Hes1*^*fl/fl*^ mice, in which *Hes1*-cKO markedly increased overall survival and decreased tumor growth, although the number of spontaneously generated tumors did not differ between WT and *Hes1*-deleted mice (Fig. 4C and Figure S6A). As this conditional *Hes1* KO using LysM deletes *Hes1* not only in macrophages but also in other myeloid lineages, including monocytes, neutrophils, and DCs, we identified the specific myeloid cell type responsible for reduced tumor growth. We isolated CD11b^+^Gr1^+^ neutrophils, CD11b^+^F4/80^+^Gr1^−^ macrophages, and CD11b^+^CD11c^+^ DCs, CD3^+^ T cells, B220^+^ B cells from tumor-bearing mice and measured their *Hes1* expression using qRT-PCR. We found that immune cells from spleens of tumor-bearing mice showed substantially reduced *Hes1* expression in neutrophils and macrophages, but not in B cells, T cells, or DCs. Interestingly, among tumor tissue-infiltrating immune cells, reduced *Hes1* expression was also observed in DCs, as well as in neutrophils and macrophages (Fig. 4D). Type-2 conventional DCs, which have macrophage-like characteristics and express CD11b, are recruited to tumor tissues [37]; therefore, it is likely that *Hes1* expression is decreased in intratumoral DCs [1]. HES1 protein levels in sorted cells were assessed via intracellular staining in TC-1 tumors (Figure S7A). We observed that HES1 expression was highest in TAMs (CD11b^+^F4/80^+^Gr1^−^). Since neutrophils (CD11b^+^Gr1^+^), and myeloid-derived suppressor cells (MDSCs, CD11b^+^Gr1^high^) were not initially distinguished, we compared HES1 expression between these populations in both the tumor and spleen of TC-1 tumor bearing mice. The analysis revealed that HES1 expression is significantly higher in neutrophils compared to MDSCs (Figure S7B). Given that MDSCs are also known to function similarly to TAMs [12], we examined HES1 protein expression in both TAMs and MDSCs from tumor, spleen, and bone marrow of TC-1 tumor bearing mice. The results showed that HES1 expression in MDSCs was significantly lower compared to TAMs (Figure S7C). These findings suggest that while various myeloid cells may contribute to tumor growth following HES1 knockout, TAMs are like to play the most pivotal role. HES1 expression was also examined in macrophages differentiated from bone marrow and spleen, as well as TAMs sorted from tumors of TC-1 tumor-bearing mice. We found that HES1 was prominently expressed only in TAMs of WT mice (Fig. 4E). In the TC-1 tumor model, in vivo administration of antibody against colony-stimulating factor 1 receptor (CSF1R), which regulates macrophage growth and differentiation delayed tumor growth (Fig. 4F, G and Figure S6B). Notably, inhibition of macrophages using the anti-CSF1R antibody was as effective in delaying tumor growth as was conditional KO of *Hes1*. After observing a comparable reduction in tumor volume between treatment with an antibody against CSF1R and the absence of *Hes1* in myeloid lineages, we reached the conclusion that the conditional knockout of *Hes1* leads to a TAM-dependent decrease in tumor volume, indicating that TAMs play a significant role in mediating the observed anti-tumor effect.

### *Hes1* ablation decreases expression of tumor-promoting factors

**Fig. 5:**
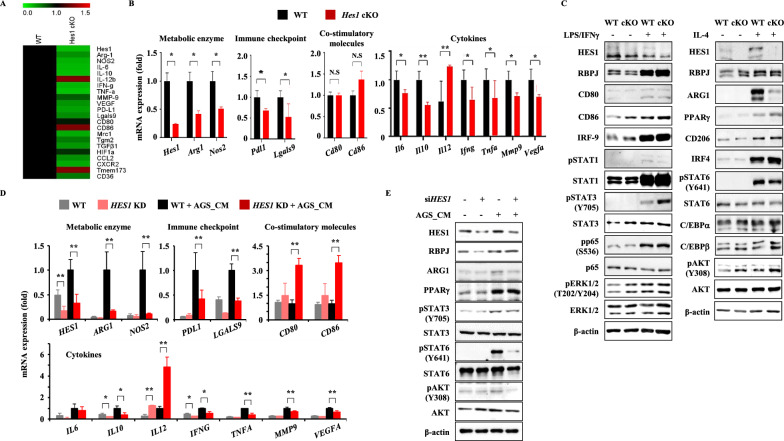
*Hes1* deficiency suppressed the expression of tumor-promoting factors. **A** Heat map showing the expression levels of TAM signature genes in WT and *Hes1*-cKO TAMs. TAMs are sorted from the TC-1 tumor model based on CD11b^+^F4/80^+^Gr1^−^ and six to eight-week-old mice were used. **B** qRT-PCR analysis of gene expression in TAMs sorted from WT and *Hes1*-cKO mice with the TC-1 tumor model, including cytokine (*Il6*, *Il10*, *Il12*, *Ifng*, and *Tnfa*), metabolic enzyme (*Arg1* and *Nos2*), immune checkpoint (*Pdl1*), co-stimulatory molecules (*Cd80* and *Cd86*), other pro-tumoral factors (*Vegfa*, *Mmp9*, and *Lgals9*). **C** BMDMs were treated with either inflammatory stimuli (100 ng/mL LPS and 20 ng/mL IFN-γ) or anti-inflammatory stimuli (20 ng/mL IL-4) for 8 h. Protein levels are analyzed by Western blotting and β-actin was used as a loading control. Non-specific bands are marked with an asterisk. **D** THP1 cells were transiently transfected with siRNA against *HES1* (at least 24 h) and treated with tumor CM from AGS cells (stomach cancers) for 8 h. qRT-PCR was conducted to measure the gene expression. **E** THP1 cells were transiently transfected with siRNA against *HES1* (at least 24 h) and treated with tumor CM from AGS cells (stomach cancers) for 8 h. Protein levels are analyzed and quantified by Western blotting. β-actin was used as a loading control

We measured the changes of gene expression in TAMs by the absence of *Hes1*. TAMs were sorted as CD11b^+^F4/80^+^Gr1^−^ from subcutaneous TC-1 tumors and RNA sequencing was performed. Since TAMs exhibit an M2-like phenotype, especially at later stages of tumor progression, high expression of pro-tumor genes is expected. As expected, conditional KO of *Hes1* reduced pro-tumor gene expression and promoted anti-tumor gene expression (Fig. 5A, Figure S8A). To further confirm the changes in gene expression caused by *Hes1* KO, TAMs were sorted again and analyzed by qRT-PCR. Gene expressions for tumor promoting genes such as *Arg1*, *Il6*, *Il10*, *Mmp9, Lgals9*, and *Vegfa* were significantly decreased, whereas that of the tumor suppressive gene such as *Il12* increased (Fig. 5B). Immune checkpoint genes such as *Pdl1* showed decreased expression in *Hes1*-cKO TAMs. Next, we checked whether the reduction in mRNA expression levels induced by *Hes1* KO led to differences in protein expression. BMDMs of WT or *Hes1*-cKO mice were treated with the M1-polarizing stimulus of LPS and IFN-γ jointly or the M2-polarizing stimulus of IL-4. The Notch signaling pathway-associated protein, RBPJ, was upregulated by both stimuli (Fig. 5C). Notably, Arginase-1 (Arg1), a cytosolic enzyme that catalyzes the hydrolysis of L-arginine to L-ornithine and urea, the expression of which is known to be induced by IL-4 and upregulated in TAMs, was substantially downregulated on *Hes1*-cKO. The expression levels and phosphorylation status of transcription factors, including IRF4, STAT6, PPARγ, C/EBPα and C/EBPβ, which are known to regulate the expression of *Arg1* [38–41], were not affected by *Hes1* KO (Fig. 5C and Figure S8B). To investigate whether *Hes1* KO altered other canonical activation pathways, BMDMs were polarized toward classical M1 or M2 phenotypes. Toll-like receptor (TLR) and mitogen-activated protein kinase signaling cascades are known to enhance inflammatory gene responses [42–45], whereas the STAT6 and mTOR pathways are known to activate anti-inflammatory responses [46, 47]. *Hes1* KO did not alter the expression patterns or activation status of these pathways in response to the polarization cues tested, as assessed by phosphorylation (Figure S8C). Similar outcomes were observed in a THP-1 cell line (Fig. 5D, E), transfected with siRNA targeting *HES1* and treated with tumor conditioned media (CM) produced by AGS cells. ARG1 was upregulated by tumor-CM, whereas *HES1* knockdown by siRNA resulted in reduced ARG1 expression. One significant distinction observed between THP-1 cells and BMDMs was a reduction in STAT6 phosphorylation upon treatment with AGS CM. It is worth noting that while BMDMs were stimulated solely with purified IL-4 as a single stimulus, THP-1 cells were exposed to tumor-CM, which is a complex mixture of various stimuli. This disparity between a purified single stimulus and the multifaceted nature of tumor-CM likely accounts for the observed outcome, highlighting the influence of the differing stimuli on the observed decrease in STAT6 phosphorylation (Figure S8D). Taken together, HES1 expression increased only when a tumor promoting stimulus was applied, and ARG1 expression also increased under the same circumstances.

### HES1 directly regulates Arg1 expression

**Fig. 6:**
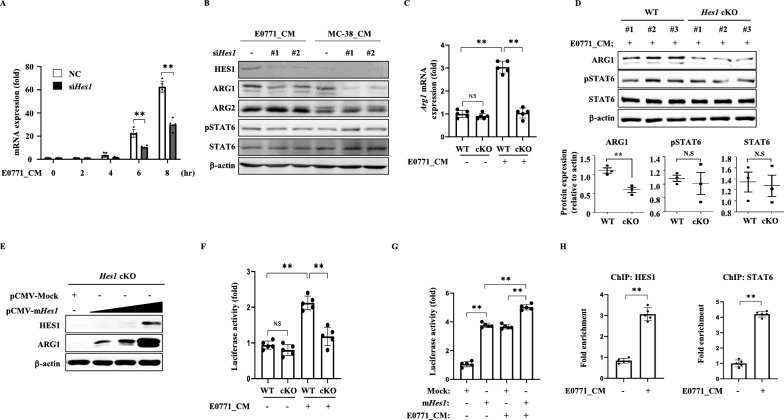
Expression of *Arg1* is regulated by HES1. **A** *Hes1*-targeted siRNA was transfected into BMDMs, which were then treated with EO771 tumor conditioned media for up to 8 h. The abundance of *Arg1* mRNA was measured using qRT-PCR. **B** BMDMs obtained from WT or *Hes1*-cKO mice were treated with either EO771 or MC-38 CM for 8 h. Western botting analyzed protein levels of HES1, ARG1, ARG2, p-STAT6 (Y641), and STAT6. β-actin was used as a loading control. **C** BMDMs were treated with EO771 CM for 8 h. mRNA for *Arg1* was measured by qRT-PCR. **D** Western blot analysis was performed to compare the expression of EO771 CM induced proteins in WT and *Hes1*-cKO BMDMs after 8 h of stimulation. The intensity of the protein bands was quantified and normalized to β-actin. **E** The effect of HES1 overexpression on ARG1 protein expression in BMDMs. BMDMs were transfected with increasing amounts of *Hes1* for 24 h and protein levels were detected by Western blots. **F** The luciferase activity was measured in BMDMs transfected with the putative HES1 binding sites on *Arg1* promoter. Wil-type or *Hes1*-cKO BMDMs were treated with EO771 CM for 8 h. **G** BMDMs were overexpressed with *Hes1* for 24 h followed by EO771 CM for 8 h. **H** RAW 264.7 cells were treated with EO771 CM for 8 h and chromatin immunoprecipitation was performed and analyzed by qRT-PCR

To confirm that *Arg1* expression is directly regulated by HES1 in TAMs, BMDMs were transiently transfected with siRNA against *Hes1* and treated with CM from EO771 tumor cells. In the control group, *Arg1* mRNA expression, which was increased by EO771 CM treatment, decreased significantly when *Hes1* was knocked down (Fig. 6A). Similarly, CM-induced protein expression of ARG1 was decreased by *Hes1* knockdown, but not ARG2 (Fig. 6B). We determined the levels of STAT6 and the corresponding phosphorylated form. It is known that STAT6 binds to the *Arg1* promoter and regulates transcription [48, 49]. However, *Hes1* knockdown did not affect STAT6 activation as assessed by phosphorylation status. We observed that *Arg1* expression increased in response to EO771 CM or IL-4 stimulation, but was significantly decreased in *Hes1*-cKO BMDMs in the presence of either stimulus (Fig. 6C and Figure S9A). This led to a decrease in ARG1 protein expression in *Hes1*-cKO BMDMs in response to CM stimulation (Fig. 6D). Even though STAT6 activation plays a key role in *Arg1* expression and STAT6 was activated by CM treatment [50], the absence of HES1 decreased ARG1 expression independent of STAT6 activation. Furthermore, *Arg1* expression was markedly reduced by *Hes1* knockdown, whereas *STAT6* knockdown also reduced *Arg1* expression, though not to the extent of *Hes1* knockdown. As expected, simultaneous knockdown of *Hes1* and *STAT6* resulted in a decrease in *Arg1* expression comparable to that seen with *Hes1* knockdown alone. Given that STAT6’s role in regulating *Arg1* expression has already been established, these findings suggest that HES1 is more potent regulator of *Arg1* expression (Figure S9B and C). We also confirmed that as the amount of transfected *Hes1* increased, ARG1 levels increased in a dose-dependent manner (Fig. 6E). We cloned the putative HES1 binding site in the *Arg1* promoter for use in a luciferase reporter assay. The HES1 binding sites in the *Arg1* promoter region were predicted using the JASPAR website [51]. Under the control conditions, *Hes1* knockout alone did not significantly affect luciferase activity. However, when BMDMs were treated with EO771 CM or IL4, an increase in luciferase activity was observed. Notably, *Hes1*-cKO resulted in a significant reduction in luciferase activity, suggesting tumor secreting factors or IL4 induced arginase1 expression that was dependent on HES1 in BMDMs (Fig. 6F and Figure S9D). Moreover, HES1 overexpression alone or with additional CM or IL4 stimulation, resulted in a significant increase in luciferase activity (Fig. 6G and Figure S9E). A chromatin immunoprecipitation (ChIP) assay was conducted to confirm that HES1 directly binds to the *Arg1* promoter. The results showed that the presence of tumor-CM significantly increased binding of HES1, along with STAT6, to the *Arg1* promoter, suggesting HES1 directly binds to the *Arg1* promoter to lead its expression (Fig. 6H). Taken together, these findings suggest a critical regulatory role for HES1 in ARG1 expression in TAMs.

### *Hes1* deficiency enhances cytotoxic T-cell infiltration into the TME

**Fig. 7:**
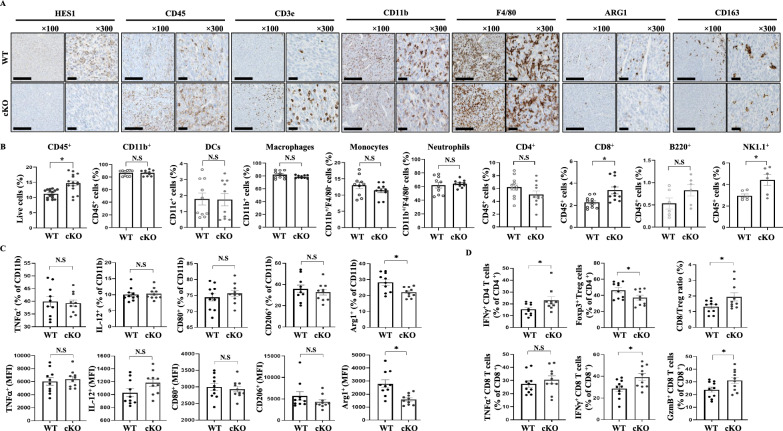
The improved TME by *Hes1* deficiency. **A** Representative IHC images (100x, 300x) showing HES1, CD45, CD3e, CD11b, F4/80, ARG1 and CD163 staining of tumors from TC-1 tumor-bearing WT and *Hes1*-cKO mice. Scale bars: 200 μm. **B** Tumors were collected from TC-1 tumor-bearing WT or *Hes1*-cKO mice and processed into single cells. Flow cytometry was performed to assess the extent of immune cell infiltration into the tumors. Representative graphs from at least three independent experiments of 3–4 animals per group are presented. The error bars represent the standard error of the mean. **C** Percentage and MFI of TNFα, IL-12, CD80, CD206, or ARG1 expression in total myeloid cells isolated from TC-1 tumors and measured by flow cytometry. **D** T cell subpopulation analyzed by flow cytometry. For ex vivo stimulation, CD4^+^ or CD8^+^ T cells from each harvested tumor were treated with Cell Activation Cocktail containing GolgiStop for 4 h

Solid tumors are frequently infiltrated by immune cells, in a manner that is closely associated with clinical outcomes [1]. Immune cells accumulate in tumor tissues and play a critical role in eliminating tumor cells or, alternately, promoting tumor growth and metastasis [10, 19, 52]. Cytotoxic T cells are particularly important for anti-tumor immunity and increasing the functional activity and number of these cells within the tumor is a crucial strategy in cancer immunotherapy [53–57]. To examine the impact of conditional KO of *Hes1* in TAMs on anti-tumor immunity, we analyzed infiltrating immune cells in subcutaneous TC-1 tumor tissues using immunohistochemistry and flow cytometry. We confirmed the infiltration of immune cells into tumor tissue by detecting CD45^+^ immune cells. Conditional KO of *Hes1* in TAMs led to a significant increase in immune cell recruitment (Fig. 7A and Figure S10A). Among the immune cells recruited, CD3^+^ T cells were a major fraction in *Hes1*-cKO tumors. Numbers of myeloid-type immune cells, particularly macrophages, which typically comprise the largest proportion of recruited immune cells in tumors, were unaffected by the *Hes1* KO. Notably, numbers of ARG1-positive immune cells were significantly reduced by the *Hes1* KO, with a decrease in the numbers of tumor-promoting TAMs that were CD163-positive macrophages (Fig. 7A). We further observed an increase in CD45-positive cells by flow cytometry, whereas other myeloid-type cells such as DCs, neutrophils, and macrophages showed only minor differences (Fig. 7B). Notably, the predominant immune cell populations that increased in CD45^+^ total immune cells were CD8^+^ T and NK cells.

Because HES1 is absent in all myeloid lineage cells that infiltrate the tumor tissues, CD11b^+^ myeloid cells were further examined to explain the cause of reduced tumor volume observed in HES1-deficiency. TNFα^+^ myeloid cells, especially pro-inflammatory macrophages, can induce tumor cell death and immune response within the tumor, and IL-12^+^ myeloid cells can stimulate IFN-α producing CD8^+^ T and NK cells. However, the numbers of myeloid cells secreting each of these cytokines did not change with *Hes1* KO (Fig. 7C). CD80 expression on myeloid cells has been associated with a pro-inflammatory phenotype and improved patient survival in certain cancers. By contrast, CD206 expression has been linked to tumor-promoting functions and poor prognosis in several types of cancers [58–61]. Although *Hes1* KO resulted in decreased ARG1 expression in total myeloid cells as confirmed by flow cytometry in Fig. 7C, alteration in myeloid cells due to HES1 deficiency did not lead to tumor volume reduction. We further investigated the effect of *Hes1*-deficiency on T cells, which can eliminate tumor cells. We observed a significant increase in type-I helper CD4^+^ T cells that produce IFN-γ, which can enhance anti-tumor immunity. Conversely, the number of regulatory CD4^+^ T cells, which suppress immune responses, decreased. Although there was no significant difference in numbers of CD8^+^ T cells producing TNF-α, there was a significant increase in numbers of CD8^+^ T cells producing IFN-γ and granzyme B. Overall, we found a higher ratio of CD8^+^ T cells to regulatory T cells, predicting better clinical outcomes in cancer patients, with *Hes1* deficiency, with an accompanying reduction in tumor size (Fig. 7D and Figure S10B). We also conducted a co-culture assay of macrophages with T cells in vitro to evaluate the growth status and the cytokine profile of the T cells in order to directly determine the impact of macrophages on T cells (Figure S11). CD4^+^ or CD8^+^ T cells isolated from wildtype spleens were stained with CellTracer violet cell proliferation kit. Proliferation of T cells was quantified by flow cytometry, revealing enhanced proliferation when co-cultured with EO771 CM or IL-4 treated *Hes1* cKO BMDMs (Figure S11A-D). Additionally, CD4^+^ or CD8^+^ T cells isolated from wildtype spleens and co-cultured with *Hes1* cKO BMDMs were found to produce significantly higher levels of IFN-γ compared to those co-cultured with wildtype BMDMs when treated with EO771 CM or IL-4 (Figure S11E and F). To assess the influence of macrophages on T cell function and reduced tumor growth in *Hes1* cKO, an ex vivo tumor killing assay was performed. Tumor cells were co-cultured with CD8^+^ T cells isolated from wildtype spleens, along with either BMDM^WT or *Hes1* cKO^. Tumor cells were stained with Annexin V and propidium iodide to determine if they were undergoing T cell-mediated apoptosis. Tumor cells co-cultured with BMDM^*Hes1* cKO^ showed fewer live cells and an increased number of early and late apoptotic cells (Figure S11G). In summary, conditional KO of *Hes1* in TAMs led to decreased ARG1 expression and improvements in the TME, as reflected by a higher ratio of cytotoxic to regulatory T cells. These changes ultimately led to a reduction in tumor size, and targeting HES1 expression may, thus, represent a useful new strategy in cancer therapy.

### Synergy of HES1 deficiency in TAM and PD-1 blockade treatment in a murine tumor model

**Fig. 8:**
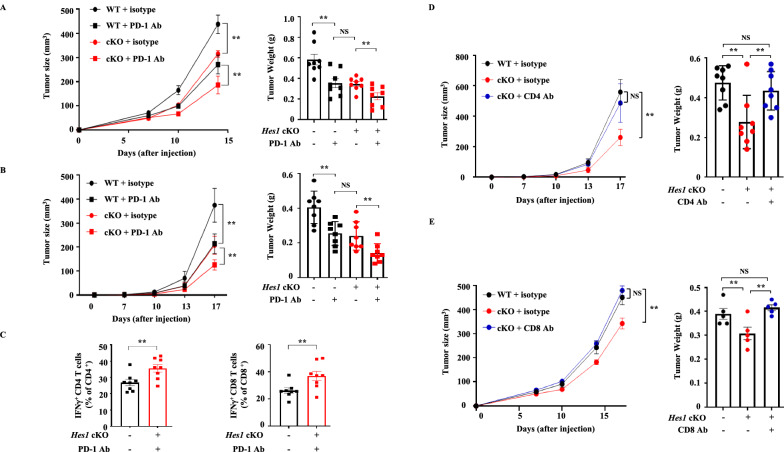
Synergistic anti-tumor effect of HES1 inhibition. **A** TC-1 cells were subcutaneously injected WT and *Hes1*-cKO mice followed by PD-1 blocking antibody. Either isotype control or α-PD1 antibodies (200 μg) was injected intraperitoneally on days 7, 10 and 13 after tumor implantations. **B** MC-38 cells were injected intraperitoneally followed by 200 μg of isotype control or α-PD1 antibodies on days 7, 10 and 13 post tumor implantations. **C** TC-1 cells were subcutaneously injected WT and *Hes1*-cKO mice followed by PD-1 blocking antibody. Either isotype control or α-PD1 antibodies (200 μg) was injected intraperitoneally on days 7, 10 and 13 after tumor implantations. IFN-γ producing CD4^+^ or CD8^+^ T cells isolated from TC-1 tumors and analyzed by flow cytometry. **D** WT and *Hes1-*cKO mice were injected subcutaneously with TC-1 cells and treated with either αCD4 or isotype control antibodies to deplete CD4^+^ T cells. Antibodies (200 μg) was injected intraperitoneally on days 7, 10 and 13 after tumor implantations. Tumor growth and weight were monitored and measured over time. **E** WT and *Hes1*-cKO mice were injected with TC-1 cells subcutaneously, and the effect of CD8^+^ T cell depletion on tumor growth and weight was evaluated. αCD8 or isotype control antibodies were administered to deplete CD8^+^ T cells. Antibodies (200 μg) was injected intraperitoneally on days 7, 10 and 13 after tumor implantations. Tumor growth curve and weight were monitored

To examine the synergistic anti-tumor effects of *Hes1*-cKO and an PD-1 immune checkpoint blockade, we utilized two subcutaneous animal tumor models (TC-1 and MC-38) and administered PD-1 blocking antibody (RMP1-14) by intraperitoneal injection. To avoid possible inefficient tumor growth inhibition by PD-1 blocking antibody when tumor growth was advanced, we administered four injections of PD-1 blocking antibody as soon as tumors started to form. We observed a notable decrease in tumor growth, both in volume and weight, in *Hes1*-cKO with PD-1 blockade when compared to WT with PD-1 blockade (Fig. 8A and B). To assess effects on immune function during tumor development, we measured immune infiltration of tumors using flow cytometry. We observed a higher infiltration of IFN-γ-producing CD4^+^ and CD8^+^ T cells in TC-1 tumors of *Hes1*-cKO mice treated with PD-1 blocking antibody, suggesting a synergistic effect in activating adaptive immunity (Fig. 8C). Based on these findings, it can be inferred that *Hes1-*cKO and PD-1 blocking antibody contributes to a slower progression of tumor growth. Next, we investigated the anti-tumor efficacy of CD4^+^ and CD8^+^ T cells with *Hes1-*cKO. Consistent with other findings, *Hes1-*cKO slowed TC-1 tumor growth; however, depletion of CD4^+^ T cells completely abolished this effect. Depletion of CD4^+^ T cells affects both regulatory CD4^+^ T cells and IFN-γ-producing CD4^+^ T cells. As illustrated in Fig. 7D, *Hes1* deficiency increased the number of IFN-γ-producing CD4^+^ T cells and decreased the number of regulatory CD4^+^ T cells. This may explain why tumors grew more aggressively with the administration of CD4-neutralizing antibody (Fig. 8D). Similarly, depletion of CD8^+^ T cells using antibodies resulted in a complete reversal of the anti-tumor effect of *Hes1-*cKO (Fig. 8E and Figure S12). Taken together, these data imply that *Hes1* on TAMs promotes tumor growth in a way that limits the activities of CD4^+^ and CD8^+^ T cells and represents a promising therapeutic approach, especially when combined with existing anti-tumor therapies.

## Discussion

Recent evidence suggests that cancer immunotherapy targeting TAMs is a promising approach [62–64]. However, a recent Phase III clinical trial of anti-CD47 antibody, magrolimab, for acute myeloid leukemia (AML) with TP53 mutation or high-risk myelodysplastic syndromes (MDS) failed to demonstrate a survival benefit. CD47 is highly expressed on cancer cells to evade phagocytosis by macrophages. Magrolimab is designed to block CD47 and help the immune system destroy cancer cells [65–67]. Despite its failure in this trial, TAMs remain an important target in cancer research due to their critical role in promoting tumor growth and progression [19]. Numerous preclinical and clinical trials have provided evidence that targeting TAMs represents an effective strategy for combating tumors. One notable example is chimeric antigen receptor (CAR)-engineered macrophages designed to target HER2 overexpressing solid tumors. CAR macrophages are resistant to immunosuppression and have a tendency for inflammation. They can not only phagocytosis cancer cells, but also help T cell adaptive immune responses by presenting tumor-derived antigens [68–70]. Based on current understanding from clinical trials, there have been relatively few trials using TAM-targeted therapies, despite the fact that TAMs make up a large proportion of the cells in the TME [10]. Preclinical studies have shown that TAMs play an important role in cancer growth suggesting that investigating tumor specific markers highly expressed on TAMs and targeting them could be a very efficient and promising approach to cancer immunotherapy.

In this study, we investigated the regulatory role of HES1, a well-established target of the Notch signaling pathway, in modulating the function of TAMs in the TME. Under normal conditions, HES1 expression is maintained at low levels in fully differentiated macrophages. However, on infiltration of the TME, expression increases in a Notch-dependent or-independent manner, suggesting a central role in defining TAM characteristics. Our findings revealed upregulation of HES1 expression in TAMs, consistent with the results of an earlier study that showed relatively higher expression of *Hes1* in monocytes and macrophages than in other immune cells in human PDCA [71]. HES1 expression was upregulated by IL-4, EGF, TGF-β, and other factors, consistent with earlier reports [72–74], including studies showing that tumor-CM contain *Hes1*-inducing factors [75, 76]. Moreover, TAMs demonstrate the capacity for self-renewal throughout tumor progression, contributing to therapeutic resistance [77]. HES1 is recognized for its role in promoting self-renewal across various cancer cell types [78–80]. Therefore, it is highly probable that HES1 plays a crucial role in the self-renewal of TAMs. Although a previous study has suggested that Notch signaling increases M1 polarization [81], there has been no report of an effect of Notch on M2 polarization to date, emphasizing the importance of studies on Notch signaling and Hes1-regulated TAM function [20, 26].

In addition, the expression of arginase-1, which converts arginine to ornithine and urea, was shown to be regulated at both mRNA and protein levels in HES1 dependent manner. Previous studies have found that TAMs upregulate expression of arginase-1 in response to pro-tumor stimuli and depletion of arginase-1 enhances the activity of T cells and promotes tumor regression [82, 83]. For this reason, arginase-1 inhibitors have been studied for their potential therapeutic applications in the treatment of cancer. INCB001158 is currently being tested for as a single agent or in combination with other therapies in patients with advanced/metastatic solid tumors [84–86]. In addition, arginase1 and 2 dual inhibitor, OATD-02 is in Phase I clinical trial to assess safety and preliminary efficacy in advanced solid tumors [87, 88]. TAMs also express high levels of ornithine decarboxylase, which converts ornithine into polyamines that promote tumor progression and impairs T-cell function [89]. It is certain that arginase activity in TAMs contributes to tumor proliferation. Interestingly, we determined that up-regulated Notch signaling, specially HES1 directly binds to *Arg1* promoter and increases its mRNA and protein expression. Reducing ARG1 expression in *Hes1-* deficient TAMs significantly increase the infiltration of IFN-γ^+^ CD4 and CD8 T into the tumors, while reducing the number of Treg cells. This alters amino acid metabolism and creates a tumor-suppressing TME. Therefore, inhibition of Notch signaling could be a good strategy to activate T cells for anti-tumor effect. However, the complete inhibition of Notch signaling to reduce arginase-1 activity is likely to affect cellular processes unrelated to tumor development. Hence, targeting macrophage expression of HES1 may be a more precise approach to restoring T-cell activity and suppressing tumor growth without interfering with other essential cellular processes.

Immune checkpoint inhibitor (ICI) therapy has shown great promise for the treatment of several types of cancer. By inhibiting checkpoint proteins including PD-1 and CTLA-4, ICIs have improved survival rates over those seen with traditional chemotherapies. However, not all patients respond well to ICIs. Therefore, understanding and improving the efficacy of ICIs is an active area of research [90, 91]. One potential explanation for the problems seen with ICI therapy is a detrimental effect of TAMs. TAMs suppress the activation and function of T cells by creating a tumor-promoting microenvironment [21]. In our study, inhibition of PD-1 in *Hes1*-depleted TAMs showed the most promising results. Tumor growth was not only significantly reduced, but this was accompanied by a significant increase in the infiltration of IFN-γ^+^ CD4 and CD8 T cells into the tumor. We conclude that depletion of *Hes1* improved the overall TME and shifted it to an anti-tumor state.

As Notch signaling plays a critical role in multiple cell types within the TME, attempts to inhibit its function should be approached with caution. For example, tumor cells rely on HES1 for self-renewal, stemness, proliferation, epithelial–mesenchymal transition, and resistance to therapy [80]. HES1 is also involved in the differentiation of regulatory T cells and contributes to the immunosuppression [92]. Considering these aspects and the findings of the present study, the development of inhibitors targeting HES1 has great potential. The results of this study, which demonstrate the improved TME in the presence of *Hes1*-depleted TAMs, provide a crucial foundation for the development of therapeutic strategies targeting TAMs.

## Conclusion

The above results indicate that HES1, a downstream target of Notch signaling, plays a critical role in TAM-mediated T cell function within the TME. Conditional knockout of *Hes1* in TAMs significantly enhanced tumor infiltration and activation of cytotoxic T cells, leading to reduced tumor growth. This study unveils a novel mechanism by which Notch signaling regulates TAM function and highlights HES1 as a promising therapeutic target for overcoming T cell suppression and potentiating cancer immunotherapy. Furthermore, the synergistic effect observed when combining *Hes1*-deficiency with PD-1 blockade warrants further investigation for the development of improved cancer treatment strategies.

## Supplementary Information

Supplementary Material 1.

Supplementary Material 2.

## Data Availability

Data is provided within the supplementary information files.

## Abbreviations

TAMs: Tumor associated macrophages
TME: Tumor microenvironment
TAF: Tumor associated factors
MDSCs: Myeloid derived suppressor cells
Treg cells: Regulatory T cells
BMDMs: Bone marrow derived macrophages
CM: Conditioned media
MMTV-PyMT: Mammary specific polyomavirus middle T antigen overexpression model
ICI: Immune checkpoint inhibitor
