# Supplementary material for: Disrupting Notch signaling related HES1 in myeloid cells reinvigorates antitumor T cell responses
Source: Exp Hematol Oncol. 2024 Dec 19;13:122. doi: 10.1186/s40164-024-00588-2 (PMC11660887; doi:10.1186/s40164-024-00588-2)
Supplement: Supplementary file 1 — Supplementary Material 1. [file 40164_2024_588_MOESM1_ESM.docx]

**Disrupting Notch signaling related HES1 in myeloid cells reinvigorates antitumor T cell responses^^[[1]](#footnote-1)^^**

Myung Sup Kim^1^, Hyeokgu Kang^1^, Jung-Hwan Baek^1^, Moon-Gyu Cho^1^, Eun Joo Chung^2^, Seok-Jun Kim^3,4,5^, Joon-Yong Chung^6^, and Kyung-Hee Chun^1, *^

^1^Department of Biochemistry & Molecular Biology, Graduate School of Medical Science, Brain Korea 21 Project, Yonsei University College of Medicine, Seodaemun-gu, Seoul 03722, Republic of Korea ^2^Radiation Oncology Branch, Center for Cancer Research, National Cancer Institute, National Institutes of Health, Bethesda, MD, 20892, USA, ^3^Department of Biomedical Science, Chosun University, Gwangju 61452, Republic of Korea, ^4^Department of Integrative Biological Sciences & BK21 FOUR Educational Research Group for Age-associated Disorder Control Technology, Chosun University, Gwangju 61452, Republic of Korea, ^5^Institute of Well-Aging Medicare & Chosun University G-LAMP Project group, Chosun University, Gwangju 61452, Republic of Korea, ^6^Molecular Imaging Branch, Center for Cancer Research, National Cancer Institute, National Institutes of Health, Bethesda, MD, 20892, USA.

*Correspondence to: Kyung-Hee Chun, Kyung-Hee Chun, Department of Biochemistry & Molecular Biology, Yonsei University College of Medicine, 50 Yonsei-ro, Seodaemun-gu, Seoul 03722, Korea. Phone: 82-2-2228-1699, E-mail: [khchun@yuhs.ac](mailto:khchun@yuhs.ac)


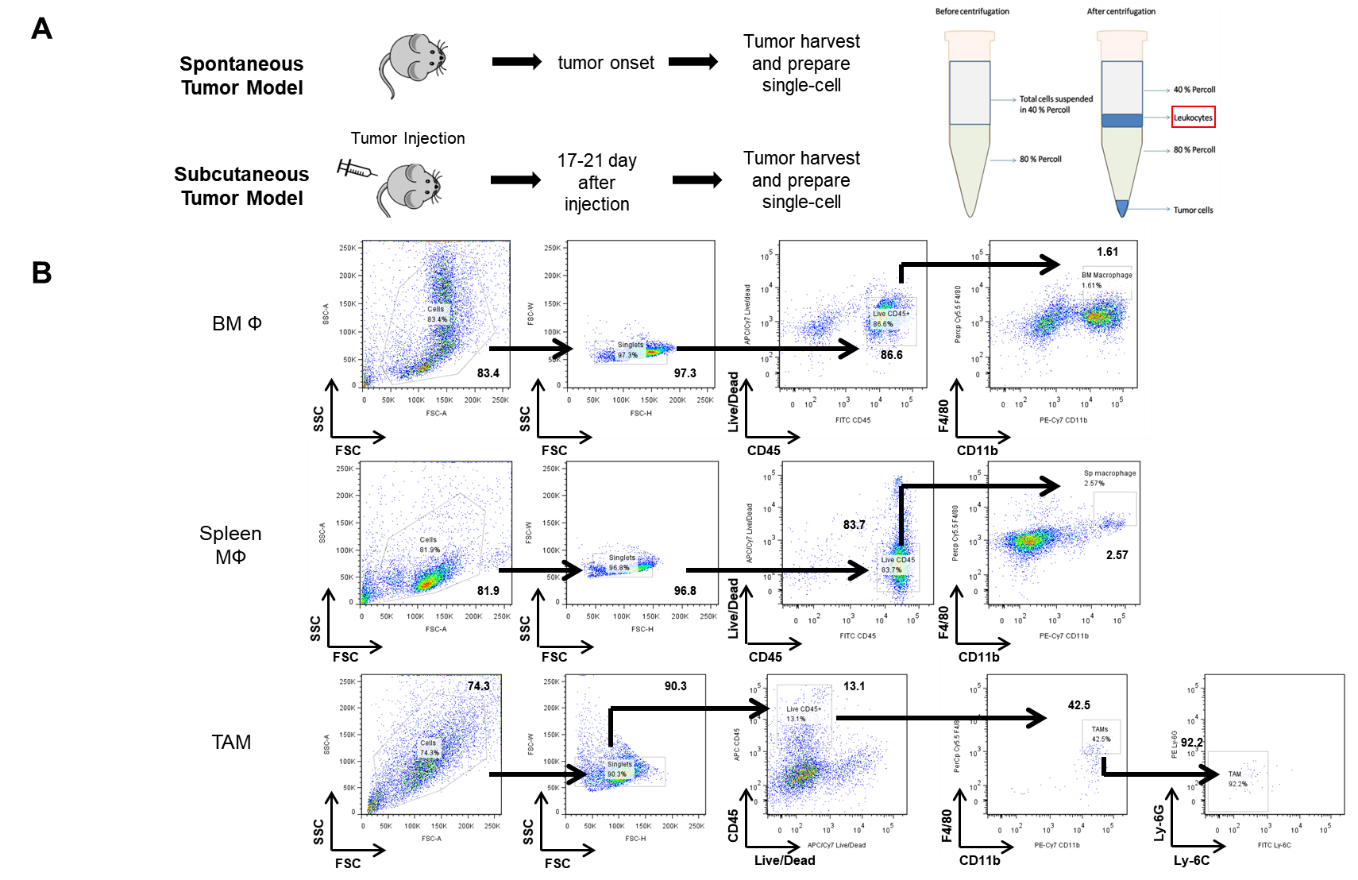


**Supplemental Figure S1. The schematic outlines of the process for isolating macrophages from bone marrow, spleen, or tumors. A.** Single cell are isolated from tumors obtained from either the spontaneous or the subcutaneous murine model and then enriched using a Percoll gradient. **B.** The gating strategy for the isolation of macrophages from bone marrows, spleen, or tumors using flow cytometry.


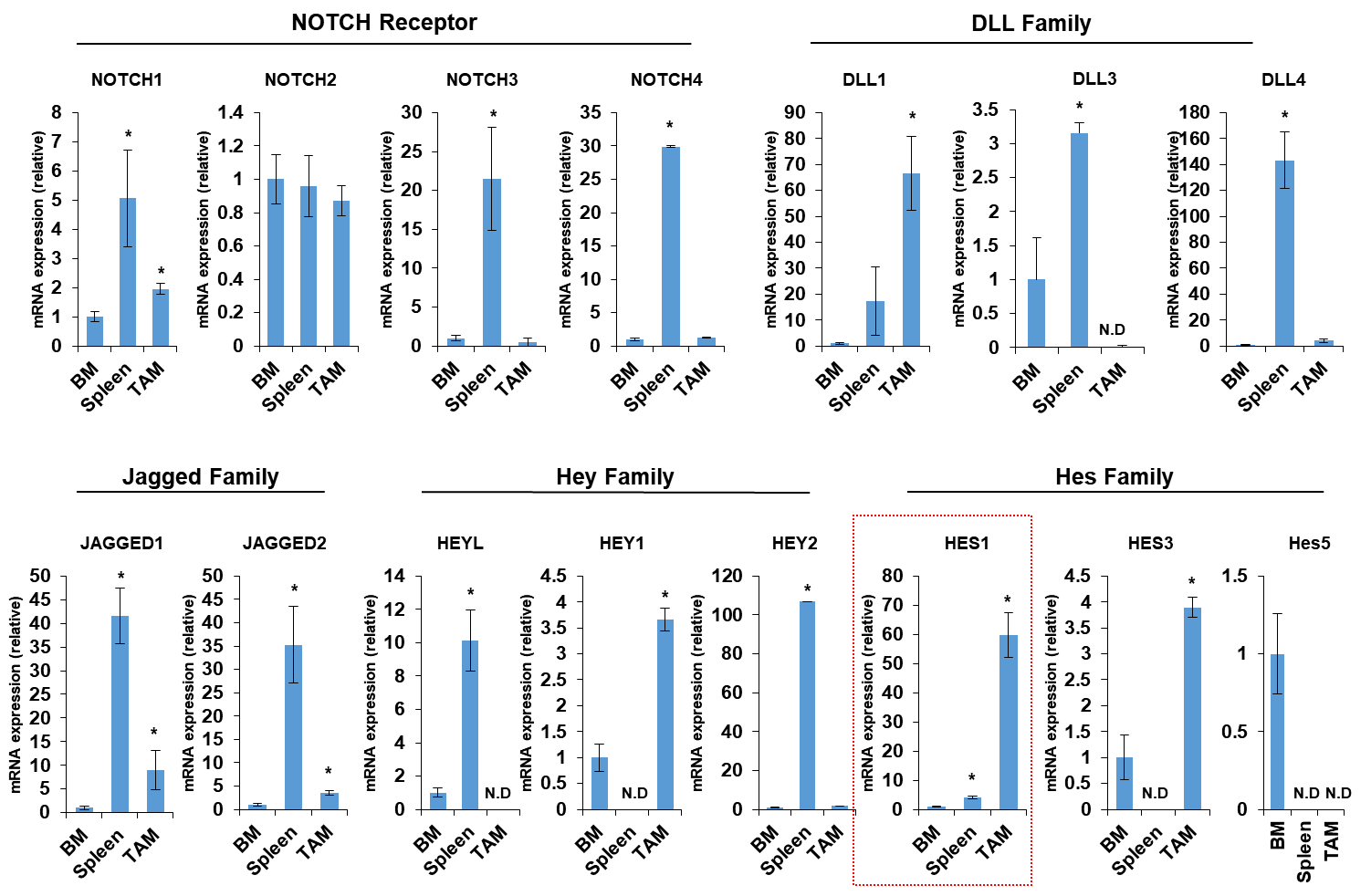


**Supplemental Figure S2 *Hes1* expression induction in macrophages sorted from tumors of MMTV-PyMT mice.** Macrophages are sorted form bone marrow, spleen, or tumors of MMTV-PyMT mice to analyze the differential expression of the genes related to Notch signaling by qRT-PCR.


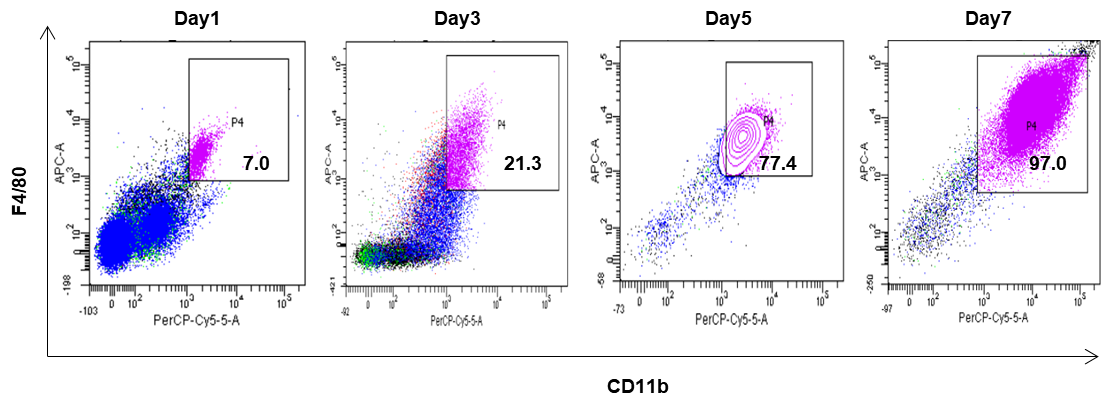


**Supplemental Figure S3 Macrophage differentiation from the bone marrow.** The bone marrow obtained from normal mice and its differentiation into BMDMs analyzed for CD11b^+^F4/80^+^ cells. The extent of differentiation to macrophages by day was quantified for Figure 1D.


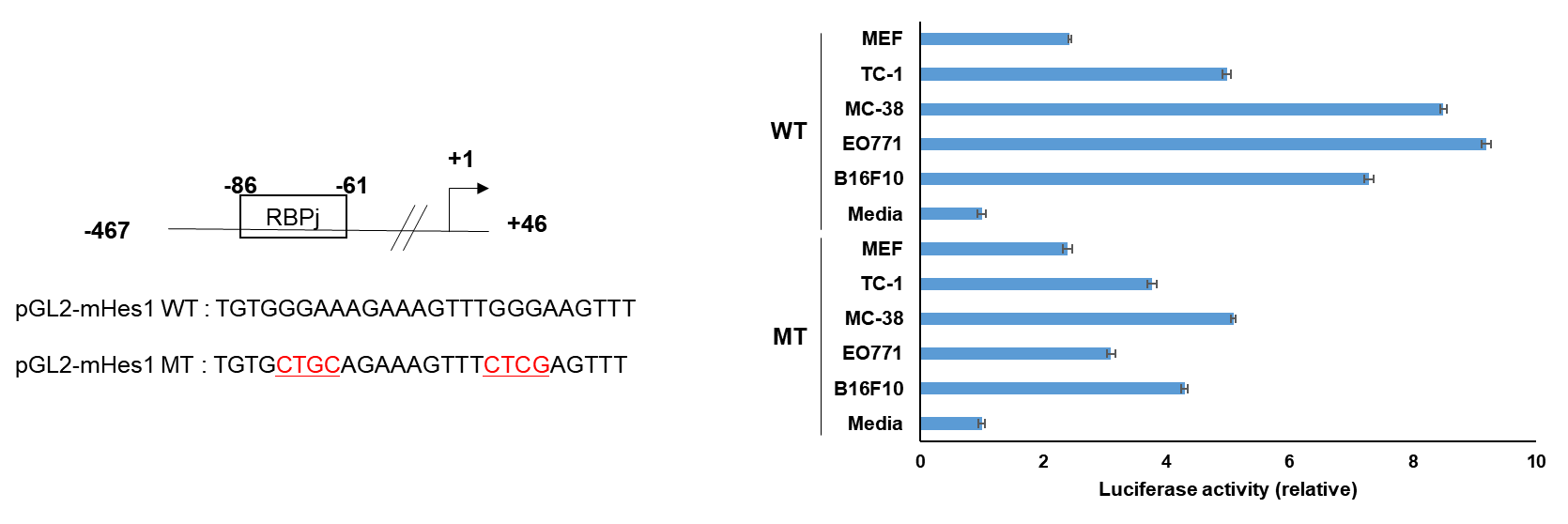


**Supplemental Figure S4 The *Hes*1 promoter sequence is depicted with either the wild-type or mutant (red) RBPJ binding sequence.** Consensus RBPJ binding motif was cloned into the pGL2 vector followed by site-directed mutagenesis on the consensus RBPJ binding sequence. BMDMs were transfected with consensus RBPJ binding motif and treated with or without conditioned media obtained from various murine tumor cell lines. Luciferase activity was normalized to the β-galactosidase activity present in the cell lysate.


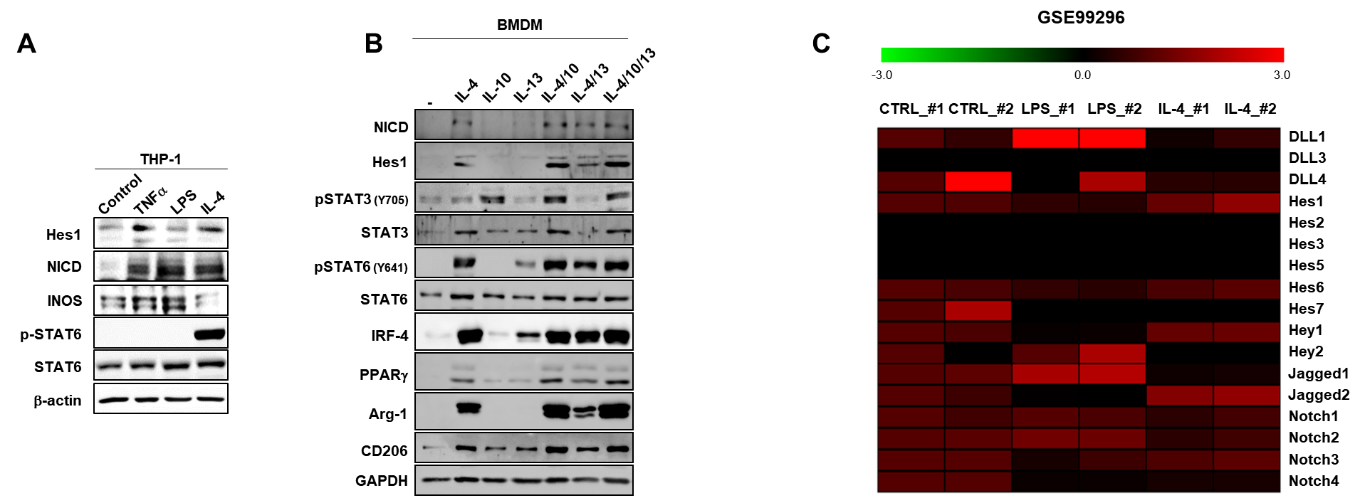


**Supplemental Figure S5 The response of HES1 expression in BMDMs to different stimuli. A.** Western blot analysis of HES1, NICD, INOS, p-STAT6 (Y641) and STAT6 in THP-1 cells stimulated with LPS (100 ng/ml), TNF-α (10 ng/ml), or IL-4 (50 ng/ml) for 18 hours. β-actin was used as a loading control. **B.** BMDMs were treated with murine recombinant IL-4 (20 ng/ml), IL-10 (10 ng/ml), IL-13 (20 ng/ml) alone or in combination for 24 hours. Protein levels were detected by Western blotting. GAPDH was used as a loading control. **C.** The expression of the Notch signaling pathway genes was analyzed using RNA sequencing data from GSE99296, which involved treating BMDMs with either LPS or IL-4.


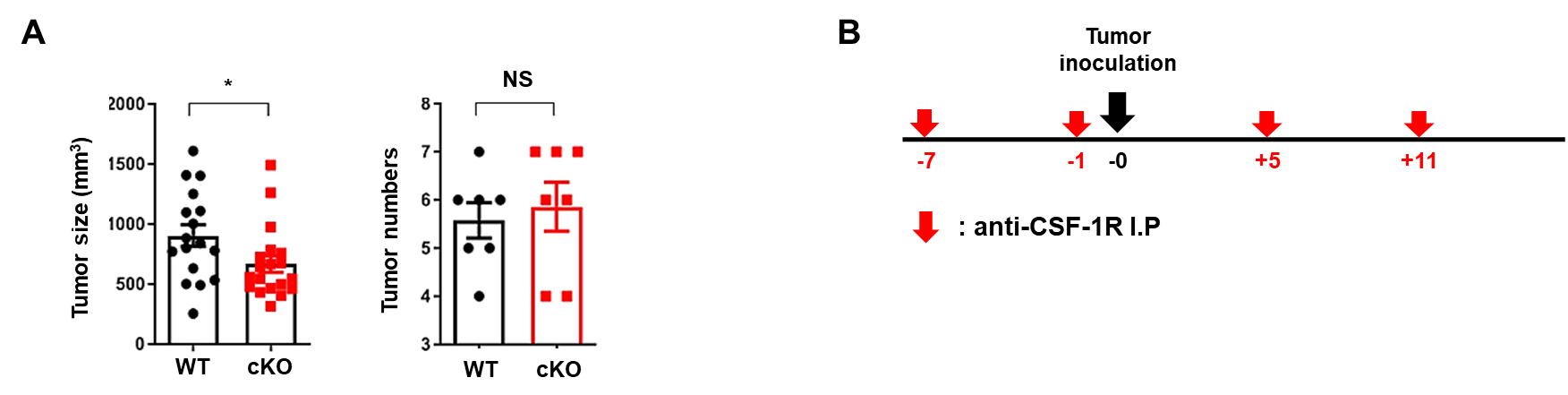


**Supplemental Figure S6 Measurement of tumor size and number of spontaneously generated tumors in the MMTV-PyMT mouse model. A.** *LysM-Hes1^+/+^* PyMT (n=4) and *LysM-Hes1^fl/fl^* PyMT mice (n=4) were used to measure the size and number of tumors. **B.** Intraperitoneal injection of α-CSF1R antibody with subcutaneous injection of TC-1 tumors.

**
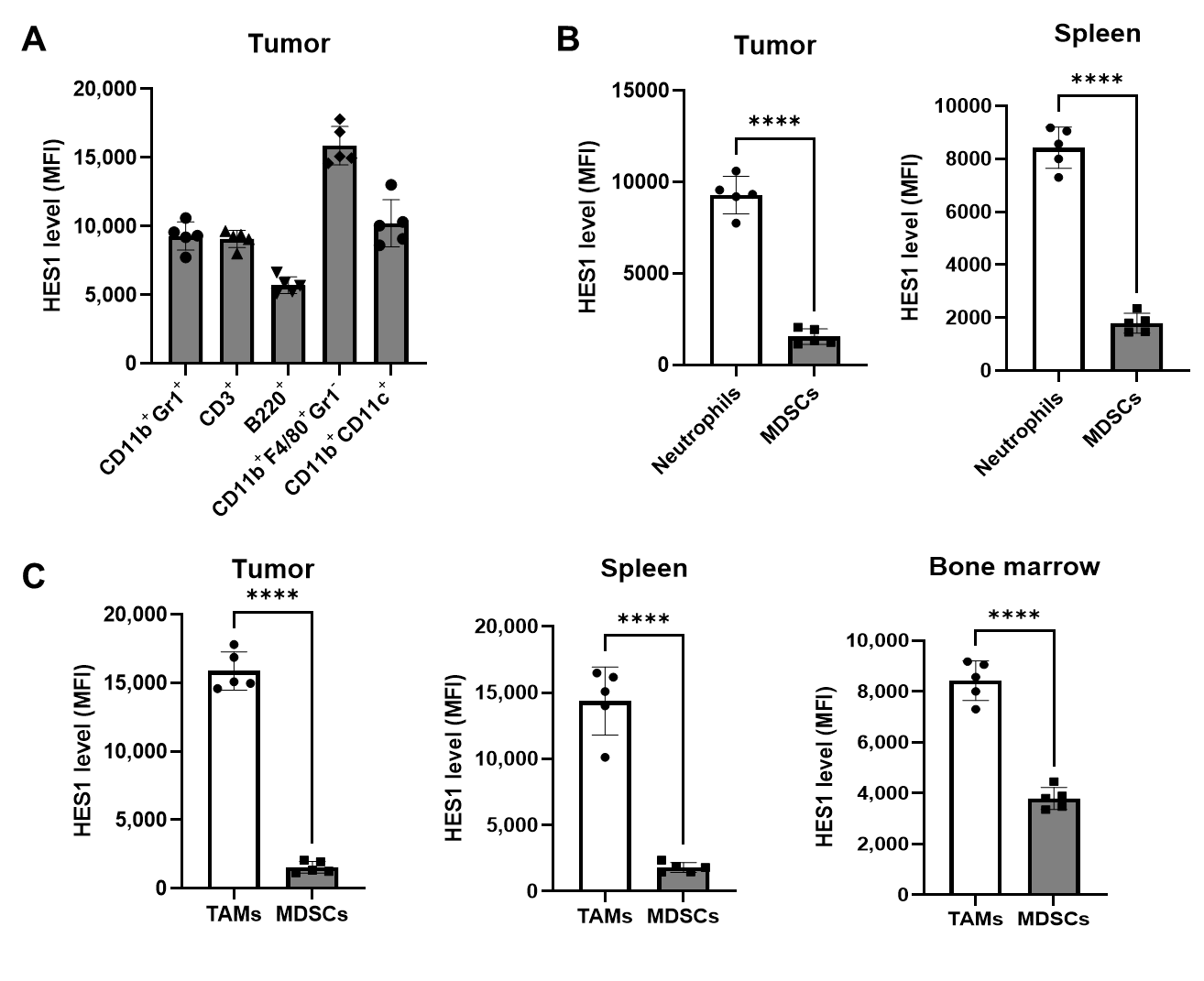
**

**Supplemental Figure S7 Measurement of HES1 protein expression in various immune cells.** **A.** WT mice were subcutaneously injected with TC-1 tumor cells. HES1 expression levels from tumor tissues are measured with intracellular staining. **B.** HES1 intracellular staining in neutrophils (CD11b^+^Gr1^+^) and MDSCs (CD11b^+^Gr11^high^) of the tumor or spleen form TC-1 tumor bearing mice. **C.** HES1 intracellular staining in TAMs (Gr1^-^CD11b^+^F4/80^+^) and MDSCs (CD11b^+^Gr11^high^) of the tumor, spleen, or bone marrow form TC-1 tumor bearing mice.


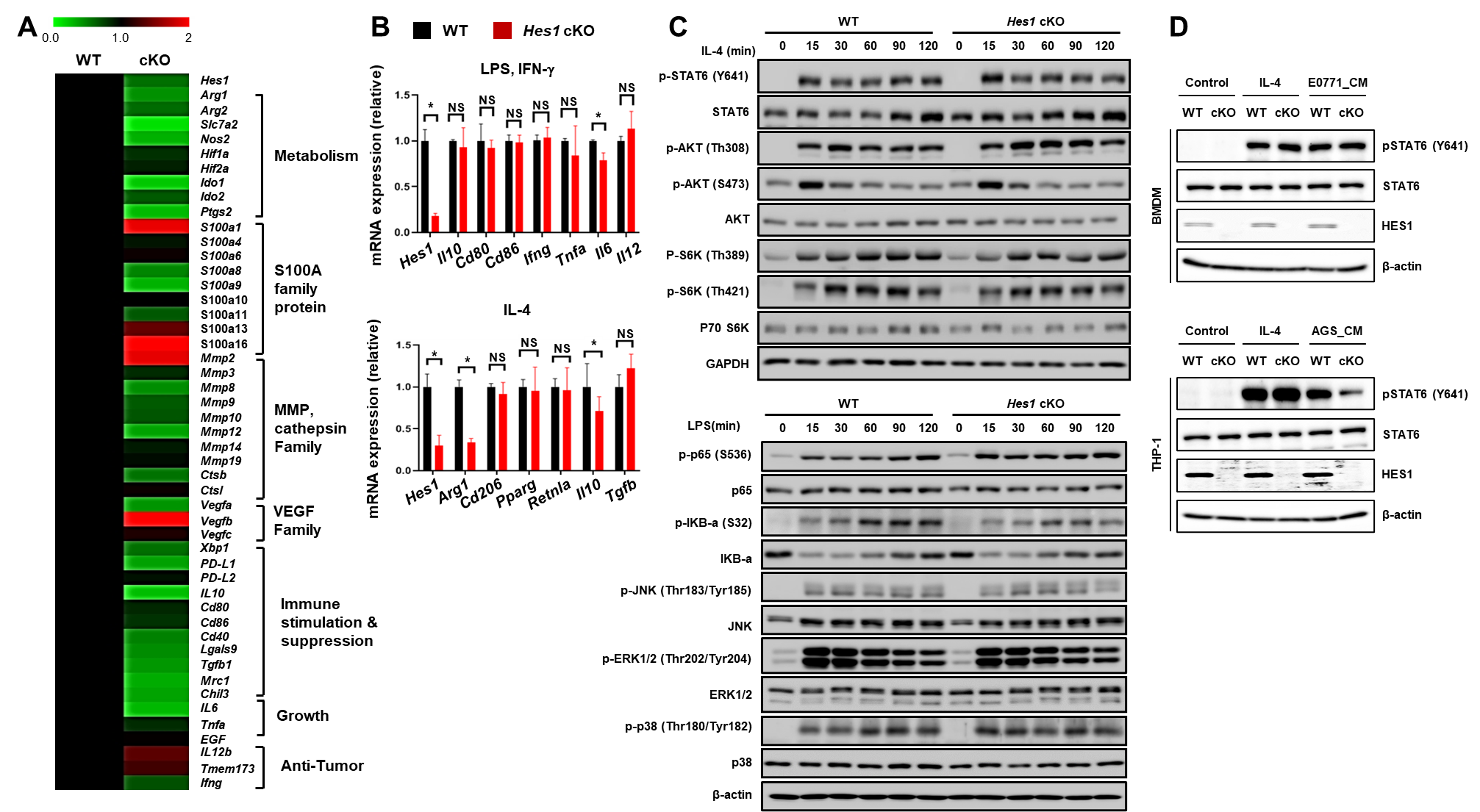


**Supplemental Figure S8 HES1 regulates TAM function. A.** Extended RNA sequencing results from Figure 5A. TAMs are sorted from WT or *Hes1* cKO mice with TC-1 tumors. Six to eight-week-old mice were used. **B.** BMDMs were treated with either inflammatory stimuli (100 ng/mL LPS and 20 ng/mL IFNγ) or anti-inflammatory stimuli (20 ng/mL IL-4) for 6 hours. Gene expression levels are analyzed by qRT-PCR and β-actin was used as a loading control. **C.** BMDMs were polarized with either 100 ng/ml LPS or 20 ng/ml IL-4 to measure canonical polarization markers. Protein levels are analyzed by Western blotting and β-actin was used as a loading control. **D.** Murine WT or Hes1 cKO macrophages are differentiated from bone marrow, whereas human macrophages, THP-1, were differentiated by PMA treatment. After differentiation, murine macrophages were treated with either 20 ng/mL IL-4 or CM for 8 hours, whereas human macrophages were transiently transfected with siRNA against HES1. THP-1 cells were treated with either 20 ng/mL IL-4 or CM for 8 hours. Protein levels are analyzed by Western blotting and β-actin was used as a loading control.


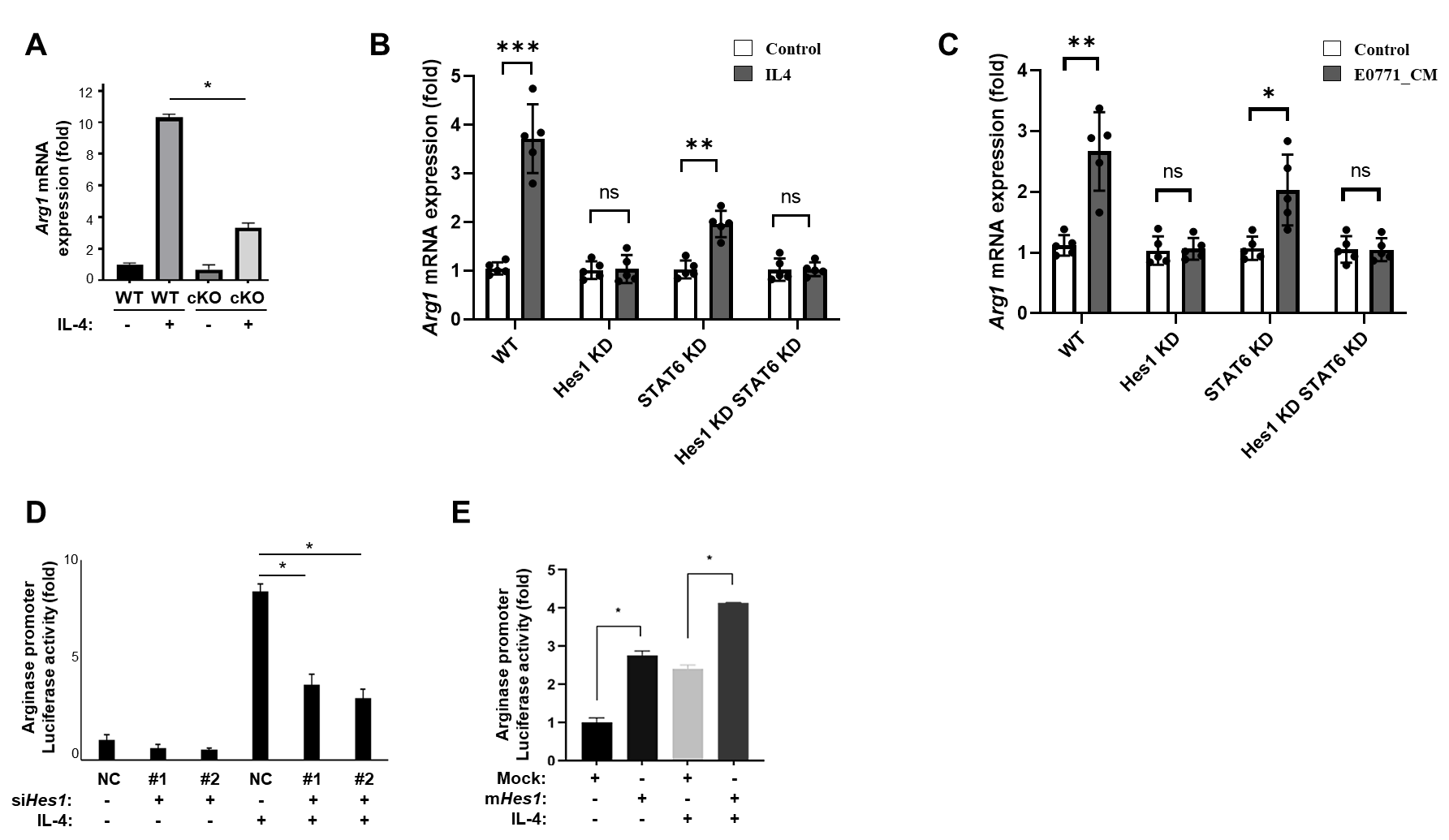


**Supplemental Figure S9 *Arg1* is regulated by HES1 in IL-4 treated BMDMs. A.** BMDMs obtained from WT or *Hes1* cKO were treated with IL-4 (20 ng/mL) for 8 hours. mRNA for *Arg1* was measured by qRT-PCR. **B and C.** BMDM WT cells were transiently transfected with siRNA against Hes1, STAT6, or both followed by IL-4 or E0771_CM treatment for 8 hours. mRNA for Arg1 was measured by qRT-PCR. **D.** The luciferase activity of BMDMs transfected with the putative HES1 binding sites on the *Arg1* promoter was measured after treating them with 20 ng/ml IL-4 for 8 hours. **E.** BMDMs were transfected with *Hes1* plasmid and incubated for 24 hours prior to treatment with 20 ng/ml IL-4 for 8 hours.


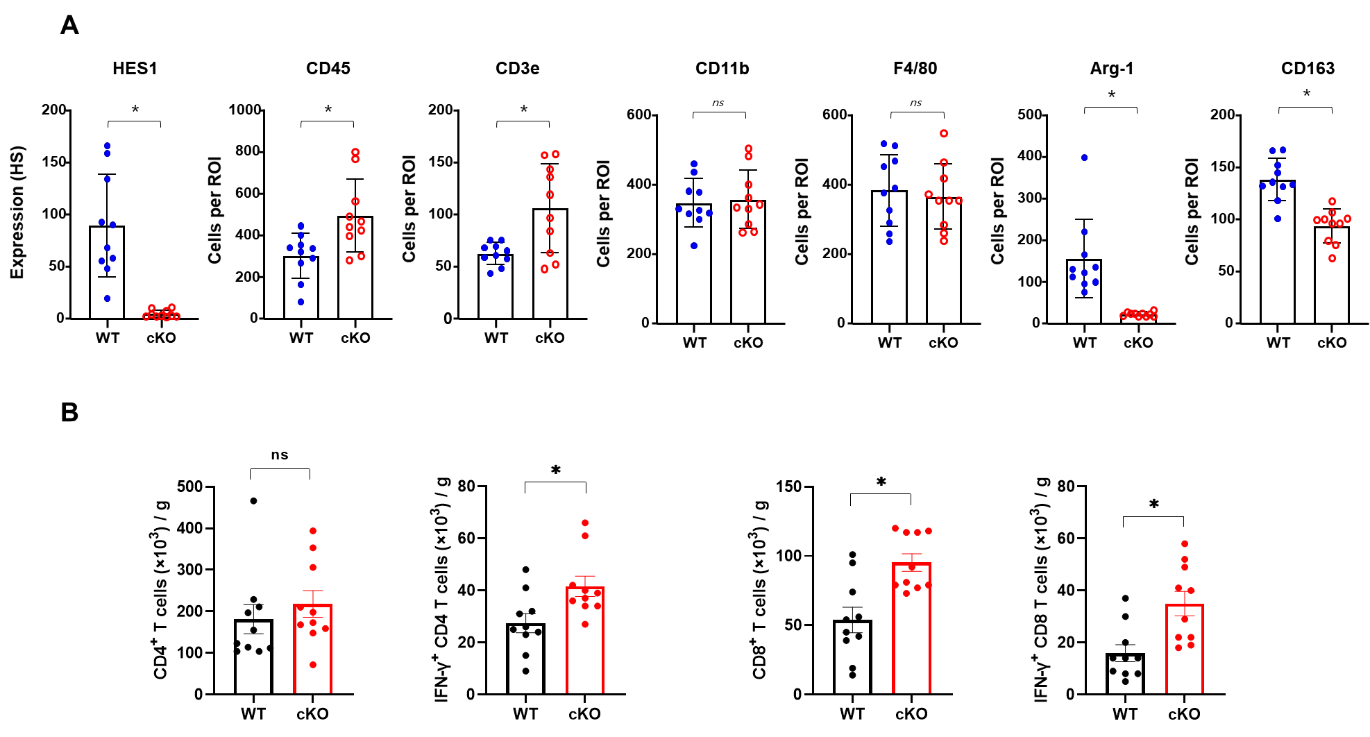


**Supplemental Figure S10 The conditional knockout of *Hes1* resulted in increased infiltration of cytotoxic T cells into the tumor microenvironment. A.** Quantified H-score of stained epitopes in Figure 7A analyzed by ImageJ. **B.** The absolute numbers of CD4^+^ T cells, CD8^+^ T cells, IFNγ^+^CD4^+^ T cells, and IFNγ^+^CD8^+^ T cells isolated from TC-1 tumors and analyzed by flow cytometry.

**
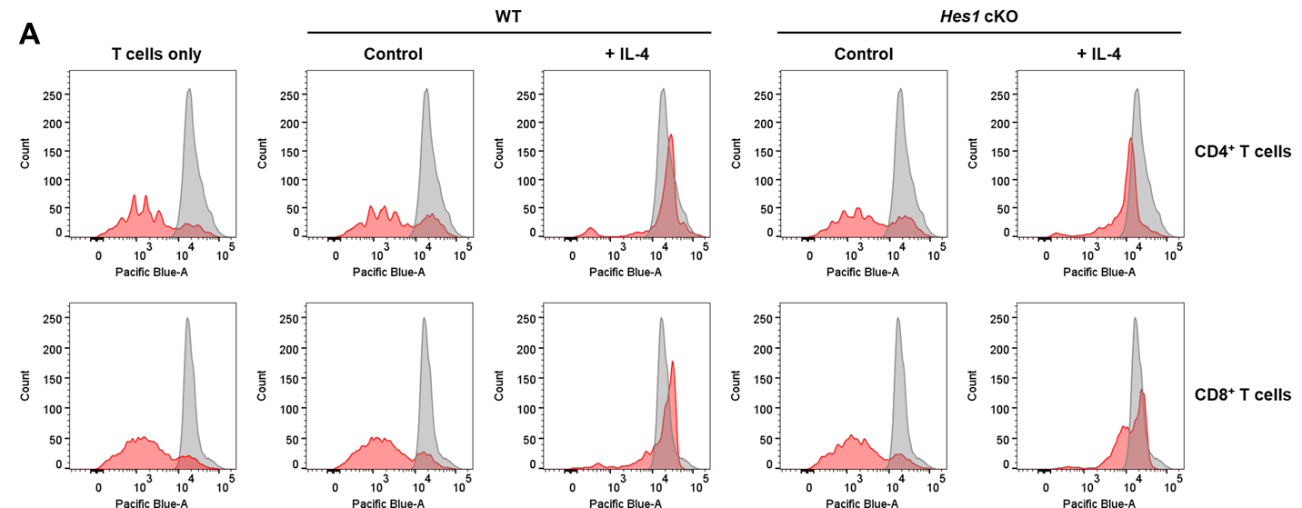
**


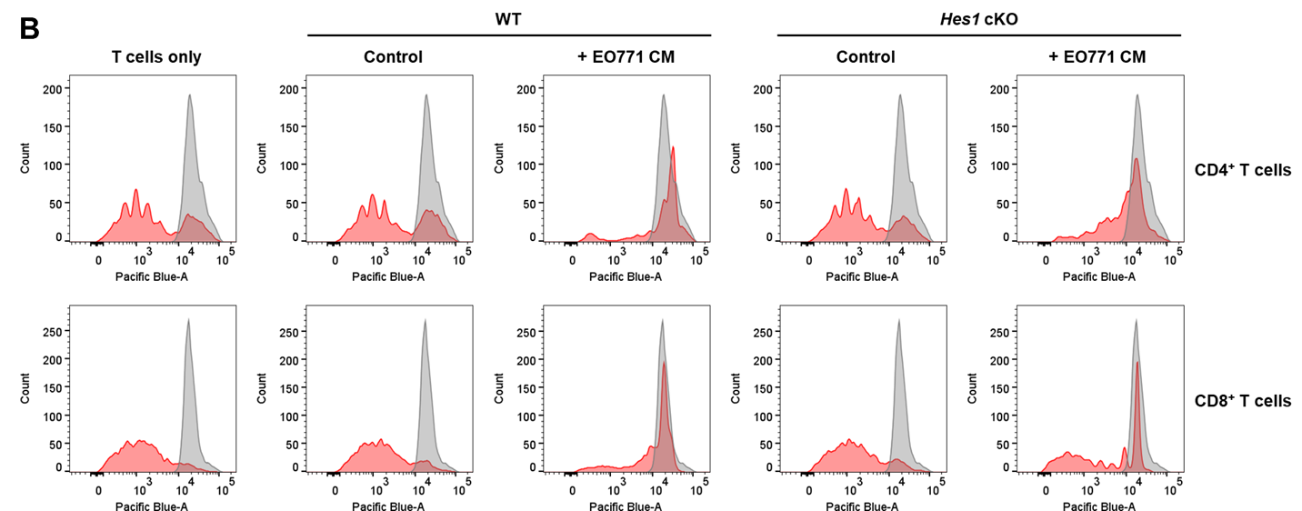


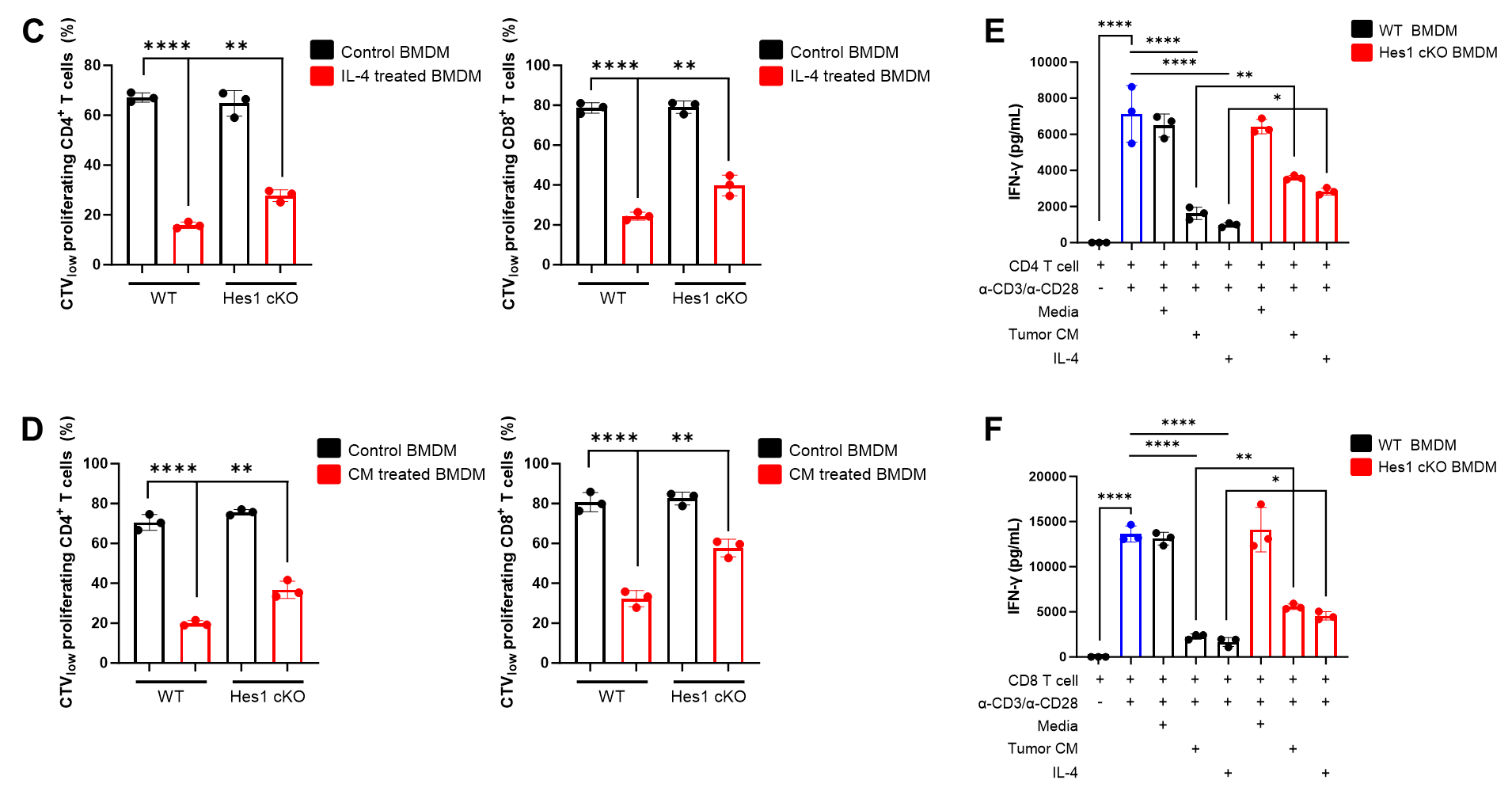
**
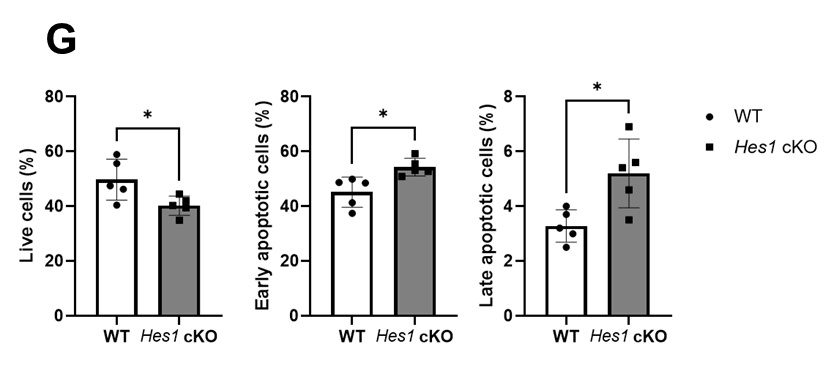
**

**Supplemental Figure S11 The Impact of macrophages on T cell growth and cytokine profiles through co-culture experiments. A.** Isolated CD4^+^ T cells or CD8^+^ T cells were stained by CellTracer^TM^ Violet (CTV) cell proliferation kit (final 5 μM). CTV labeled WT CD4^+^ or CD8^+^ T cells were left unstimulated (grey) or were stimulated using plate-bound anti-CD3/CD28 antibodies (red). T cells were co-cultured alone (T cells only) or IL-4 (20 ng/mL) treated BMDM. Cells were harvested after three days, stained and analyzed for CD4^+^ or CD8^+^ T cells by flow cytometry. Histograms indicate the CTV profile of gated CD4^+^ or CD8^+^ T cells. **B.** T cells obtained and activated in the same manner were treated with the conditioned media from EO771 breast tumor cell lines. **C.** Quantification of the results obtained in A. **D.** Quantification of the results obtained in B. **E and F.** Wildtype CD4^+^ or CD8^+^ T cells were stimulated with plate-bound anti-CD3/CD28 antibodies and co-cultured with IL4 or EO771 CM treated BMDMs from WT (black) or *Hes1* cKO (red) mice at a cell ratio of 1:3 (0.3 x 10^4^ BMDM: 1 x 10^5^ T cells). IFN-γ secretion of CD4^+^ or CD8^+^ T cells were analyzed by ELISA. **G.** Murine tumor cells (TC-1, the most frequently used cell line for animal model in this paper) were co-cultured with BMDMs differentiated from WT or Hes1 cKO mice and T cells isolated from WT spleens (CD8a^+^ T cell isolation kit, mouse, Miltenyi Biotec, 130-104-075). T cells were activated and cocultured with BMDMs and TC-1 cells at a cell ratio of 1:3:3 [BMDM (0.3 x 10^4^): T cells (1 x 10^5^): TC-1 cells (1 x 10^5^)] for 3 days. CD45 (TIL) MicroBeads (Miltenyi Biotec, 130-110-618), CD8a^+^ T cell isolation kit (Miltenyi Biotec, 130-104-075), F4/80 MicroBeads UltraPure, (Miltenyi Biotec, 130-110-443) were used to remove T cells and BMDMs from the samples. The remaining TC-1 cells were stained with FITC Annexin V (BioLegend, 640945) and propidium iodide (Merck, P4170) for 30 min in calcium containing staining buffer and analyzed by flow cytometry.


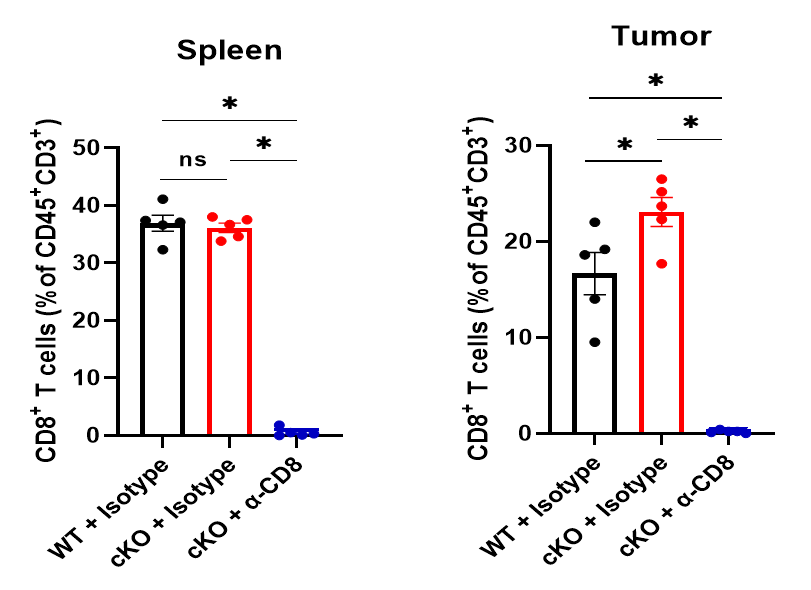


**Supplemental Figure S12 Depletion of CD8^+^ T cells by αCD8 antibodies from spleen and TC-1 tumors.** Anti-CD8 antibodies effectively deplete CD8^+^ T cells from spleens or tumors of TC-1 tumor-bearing mice.

1. Authorship notes: Myung Sup Kim and Hyeokgu Kang are co–first authors. [↑](#footnote-ref-1)
